# Adaptive and resilient protection coordination in smart microgrids enabled by a directional fault current limiter

**DOI:** 10.1371/journal.pone.0356375

**Published:** 2026-08-19

**Authors:** I. M. Shindy, Aymen Flah, Mostafa G. Rabea, Said Elmasry, Adel A. El Samhay, M. M. R. Ahmed, A. M. Hamada

**Affiliations:** 1 Electrical Power and Machine Department, Faculty of Engineering, Capital University, Cairo, Egypt; 2 Applied Science Research Center, Applied Science Private University, Amman, Jordan; 3 ENET Centre, CEET, VSB-Technical University of Ostrava, Ostrava, Czech Republic; 4 College of Engineering, University of Business and Technology, Jeddah, Saudi Arabia; 5 Department of Electrical Technology, Faculty of Technology and Education, Capital University, Egypt; 6 Railway and Modern Transportation Technology Program, Faculty of Industrial and Energy Technology, Borg Al Arab Technological University (BATU), New Borg El Arab Alexandria, Egypt; Aalto University, FINLAND

## Abstract

Power systems, particularly distribution networks, are currently facing increasing challenges due to rising energy demand and the growing complexity of modern grids. The integration of Distributed Generators (DGs) within Microgrids (MGs) introduces additional fault current contributions, which significantly modify short-circuit levels. This change can result in miscoordination problems in existing Overcurrent Relay (OCR)-based protection systems, including both maloperation and delayed tripping. In addition, the increased fault current contribution from MGs may exceed the design limits of equipment such as circuit breakers (CBs), exposing them to higher electrical stress and increasing the risk of failure to operate properly. Consequently, these issues collectively degrade the overall reliability and security of the power system. To overcome these challenges, installing a Directional Fault Current Limiter (DFCL) between the microgrid and the upstream network is considered an effective mitigation approach. The DFCL helps in controlling fault current levels and improving protection coordination. However, determining the optimal impedance setting of the DFCL is a complex task, as it must simultaneously ensure proper coordination among OCRs and reduce short-circuit levels to enhance system reliability. In this context, this paper proposes an analytical methodology for determining the optimal DFCL impedance based on Thevenin equivalent impedance analysis. The proposed approach provides a straightforward impedance sizing procedure that restores overcurrent relay coordination following distributed generation integration while maintaining system reliability. Unlike optimization-based methods, the proposed analytical approach determines the required DFCL impedance directly from the equivalent Thevenin impedance without iterative computation, making it computationally efficient and suitable for real-time engineering application The methodology is validated under different operating scenarios involving synchronous generators, wind turbine generators, and photovoltaic systems. The results demonstrate that the proposed approach effectively restores relay coordination, limits excessive fault currents, and enhances the reliability of microgrid protection without requiring adaptive protection schemes or additional relay installation.

## 1. Introduction

**Fig 1:**
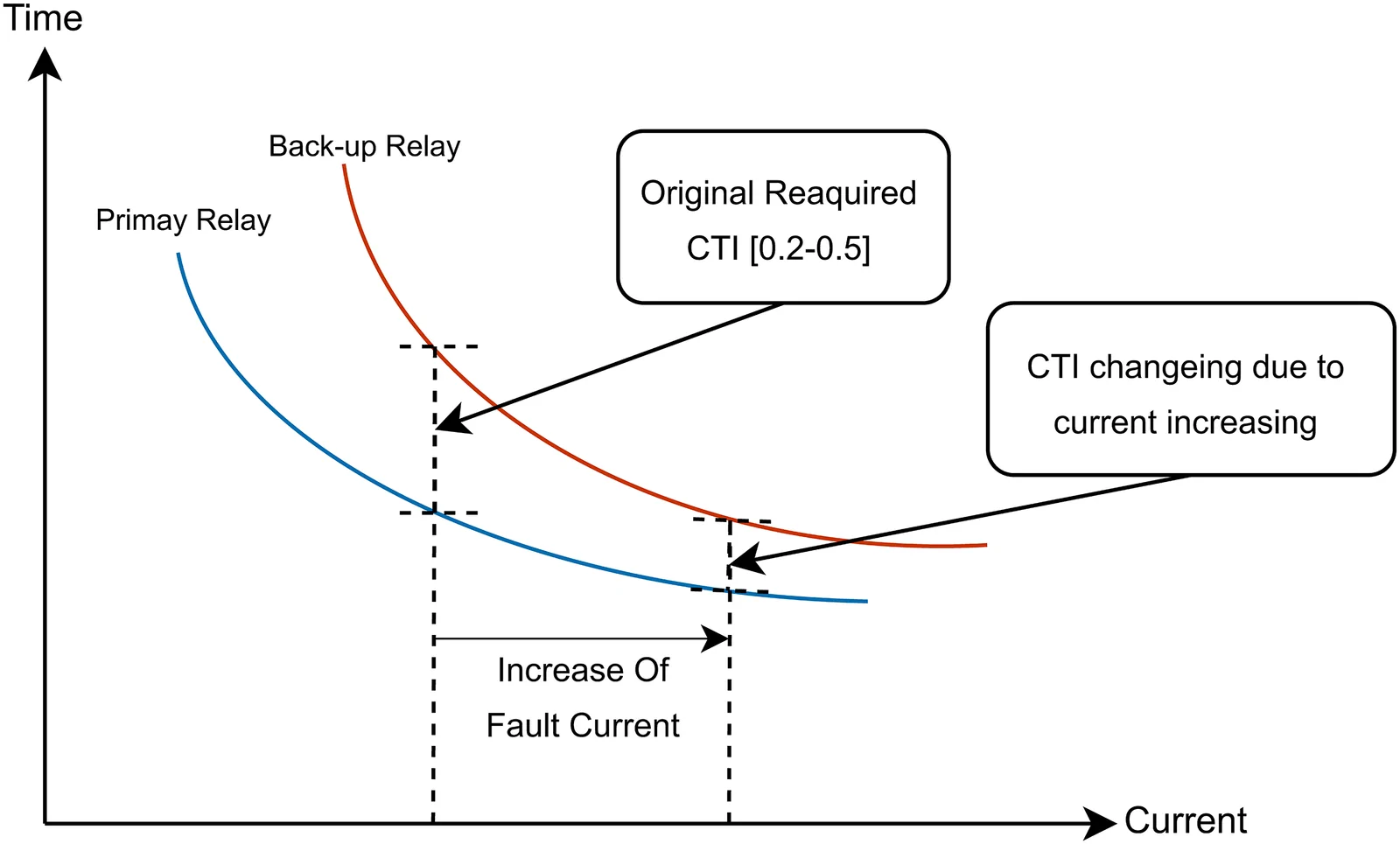
Impact of DG on coordination characteristics between primary and backup relays.

Renewable energy resources have become A microgrid is a small-scale electrical distribution system that includes different types of distributed generators, such as synchronous, induction, and inverter-based units [1], as well as EV charger [2,3] and various electrical loads. Normally, a microgrid operates connected to the main utility grid at the Point of Common Coupling. However, it can also operate independently in what is known as islanded mode. In islanded mode, the microgrid is completely separated from the main utility grid and functions on its own. One of the key features of a microgrid is its ability to disconnect from the utility grid during disturbances such as faults or voltage drops. It can also be intentionally disconnected if there are power quality issues. In both cases, the microgrid continues to supply power to local loads by operating autonomously in islanded mode. The ability of MG to operate in both grid-connected and islanded modes improves the quality of service and increases the overall reliability of the power system [4–6]. However, despite these advantages, microgrids also introduce several challenges. The integration of DGs, together with the different operating modes of the microgrid, can affect system operation, control, protection, and reliability. Therefore, these challenges must be carefully studied and properly addressed before implementing a microgrid. [7,8] If these issues are not properly addressed, the expected MG will be limited, leading to reduced system reliability. The most affected part is the protection system in the distribution network, as distribution networks are usually designed as radial systems, and their overcurrent protection schemes are coordinated without considering the presence of DG. As shown in Fig 1, Adding DGs can change the fault level in the microgrid. It can also affect the magnitude and direction of current, which are key factors in protection relay coordination. As a result, these changes may lead to miscoordination in the existing protection system [3] On the other hand, the contribution of the MG during faults, due to the response of DGs, can increase the total fault current [9] beyond the designed limits of distribution equipment such as cables and CBs. As a result, circuit breakers are exposed to higher stress levels, which increases the risk of improper operation and may also lead to protection coordination problems [10,11].

In this study, different levels and types of DGs are considered to assess their impact on fault current levels and to mitigate their negative effects [12,13]. The contribution of MG during fault conditions can lead to protection miscoordination, which may delay the operation of certain relays; as a result, fault current may flow through CBs for a longer duration.

This increases the possibility of exposing hidden failures in CBs during the isolation of the faulty section. Such failures, caused by excessive stress on the circuit breaker, are referred to as hidden CB failures [14].This may lead to malfunctions in the protection system. Therefore, if appropriate mitigation measures are not implemented, the MG may fail to deliver its expected benefits, such as enhanced reliability. Furthermore, a failure of a CB can result in multiple or cascading outages within the power system, thereby further degrading overall system reliability. To address the protection challenges associated with the integration of DG and MG operation, effective protection schemes [15] and scenarios are required to ensure reliable performance in both grid-connected and islanded modes. In this context, various protection methods and coordination techniques for microgrids have been widely investigated in recent studies [16]. In general, the proposed protection approaches can be classified as innovative schemes, where conventional protection methods primarily based on overcurrent are replaced by advanced strategies or modern protection devices [17,18].

### 1.1. Real-time protection

Adaptive protection constitutes a primary scheme within the initial category, functioning as an online and real-time process that adjusts the settings of protection relays according to the state of the MG, which includes factors such as topology, generation, and load level. This adjustment can also be achieved through the utilization of numerical relays, alongside advanced communication architectures and protocols, which are essential technical prerequisites for the effective implementation of these schemes. In this context, various other communication-based methodologies have been introduced in [19,20], Although the implementation of these innovative schemes can alleviate the challenges associated with MG protection, the associated costs and complexities are among the criticisms currently directed at them, a key concern with implementing these new protection schemes is the reluctance of utility companies to change existing relay settings or replace traditional relays with new ones. This delay in decision is especially noticeable in smart microgrids, which differ from conventional microgrids by using digital control systems, real-time monitoring, advanced communication, and automation to improve electricity generation, distribution, and consumption within a specific area such as a campus, industrial site, or community, the real-time monitoring refers to the continuous observation of electrical quantities such as voltage, current, and power flow, as well as fault indicators. It also includes detecting abnormal conditions like overcurrent, voltage drops, and frequency changes. In this paper, the flexibility of smart grid technology in responding to system changes is utilized to develop an adaptive protection method. So, this approach does not require changing relay settings or replacing existing protection devices.

### 1.2. Importance of FCL

On the other hand, these online methods are not particularly effective in mitigating the risk of damage or failure of circuit breakers, moreover, advances in power electronic-based technologies, including solid-state transformers and virtual power plants, have accelerated the transition toward inverter-dominated power systems, providing enhanced operational flexibility while introducing new challenges for system stability and protection [21]. Taking into account that it is more advantageous to rely on existing relay to address the protection challenges posed by DG integration, especially when a portion of a distribution network is transitioning to a microgrid. Another significant approach to addressing the protection challenges in distribution networks equipped with DGs and MGs involves the use of FCLs. Additionally, given that replacing CBs with higher capacity units incurs substantial costs and technical challenges, the utilization of FCLs to manage the rise in short-circuit currents has emerged as a particularly promising solution [20,22–27]. Through the application of FCL, excessive currents during a fault condition are restricted by the swift rise in FCL impedance. Conversely, during standard operational conditions, FCL remains nearly imperceptible by presenting minimal impedance.[11]. The installation of FCL not only regulates fault currents to levels compatible with the design specifications of network equipment but also facilitates the restoration of coordination among overcurrent relays. In all previously referenced cases, the FCL responds to faults occurring on both the upstream network and the MG side, irrespective of the fault current's direction.

The operation of the FCL is particularly advantageous when faults arise on the upstream side, as it helps to alleviate the adverse effects of DGs and microgrids on the protective systems of the utility network [20], however, in the event of faults on the MG side, the FCL's operation significantly diminishes the contribution from the main grid, which may result in a loss of coordination between the upstream relays and the MG relays, It is evident that under such conditions, the system's reliability may get worse, or the costs associated with maintaining reliability may escalate. To address this challenge, the implementation of a DFCL, which has been experimentally developed in [28], so referred to as DFCL, it is advisable to install this device at the PCC. The DFCL is specifically engineered to function in response to external faults occurring within the upstream network. So, the implementation of the DFCL at the PCC modifies the fault current and all contributions from the downstream network, which may influence the coordination levels among relays, potentially leading to a deterioration in overall system reliability.

## 2. Three-phase SICFCL configuration and principle of operation

**Fig 2:**
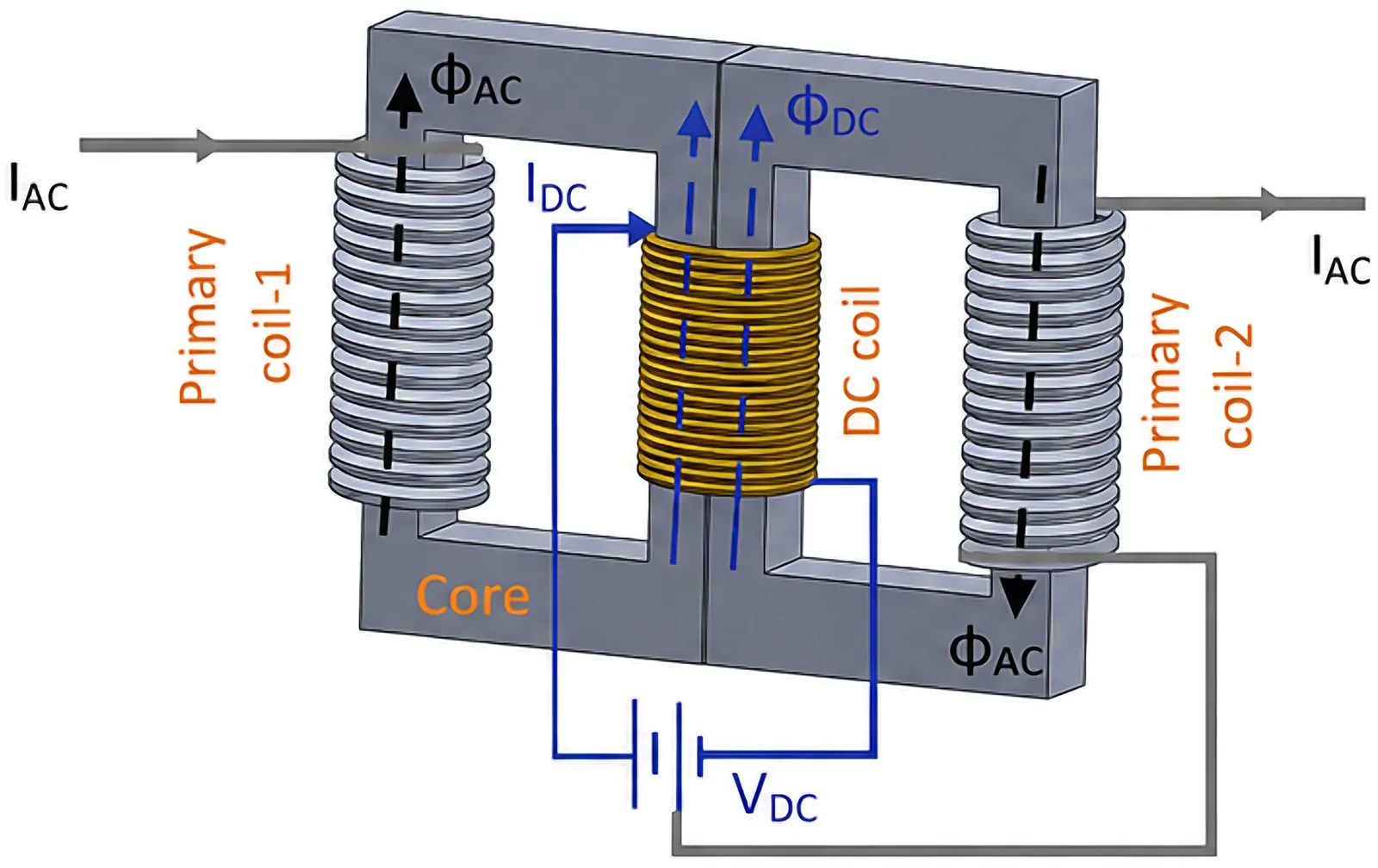
Traditional single-phase SICFCL.

Single-phase SICFCLs operate by exploiting the nonlinear magnetic permeability characteristics of the silicon steel core to suppress fault currents. Their operating mechanism is illustrated in Fig 2 Under normal operating conditions, the applied DC MMF maintains the magnetic core in a saturated state, resulting in a low impedance that minimizes the voltage drop across the device.

**Fig 3:**
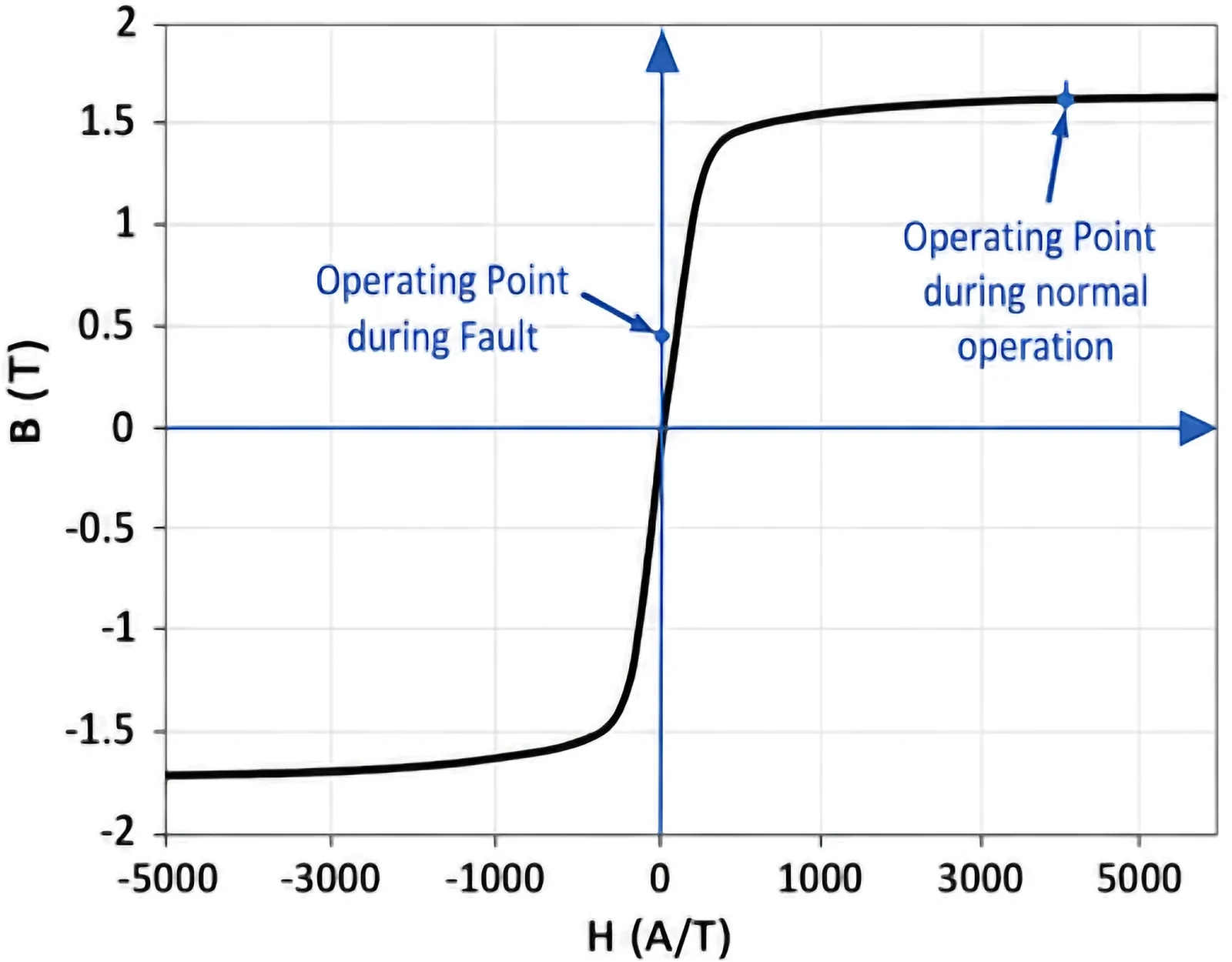
SICFCL operating points of the core.

When a short-circuit fault occurs, the fault current increases significantly, forcing the magnetic core to leave the saturation region, as shown in Fig 3. This transition causes a rapid increase in the device impedance, thereby limiting the magnitude of the fault current. The magnetic behavior presented in Fig 3 is based on the characteristics of a commercially available M470 silicon steel E-core with low manufacturing cost [29].

**Fig 4:**
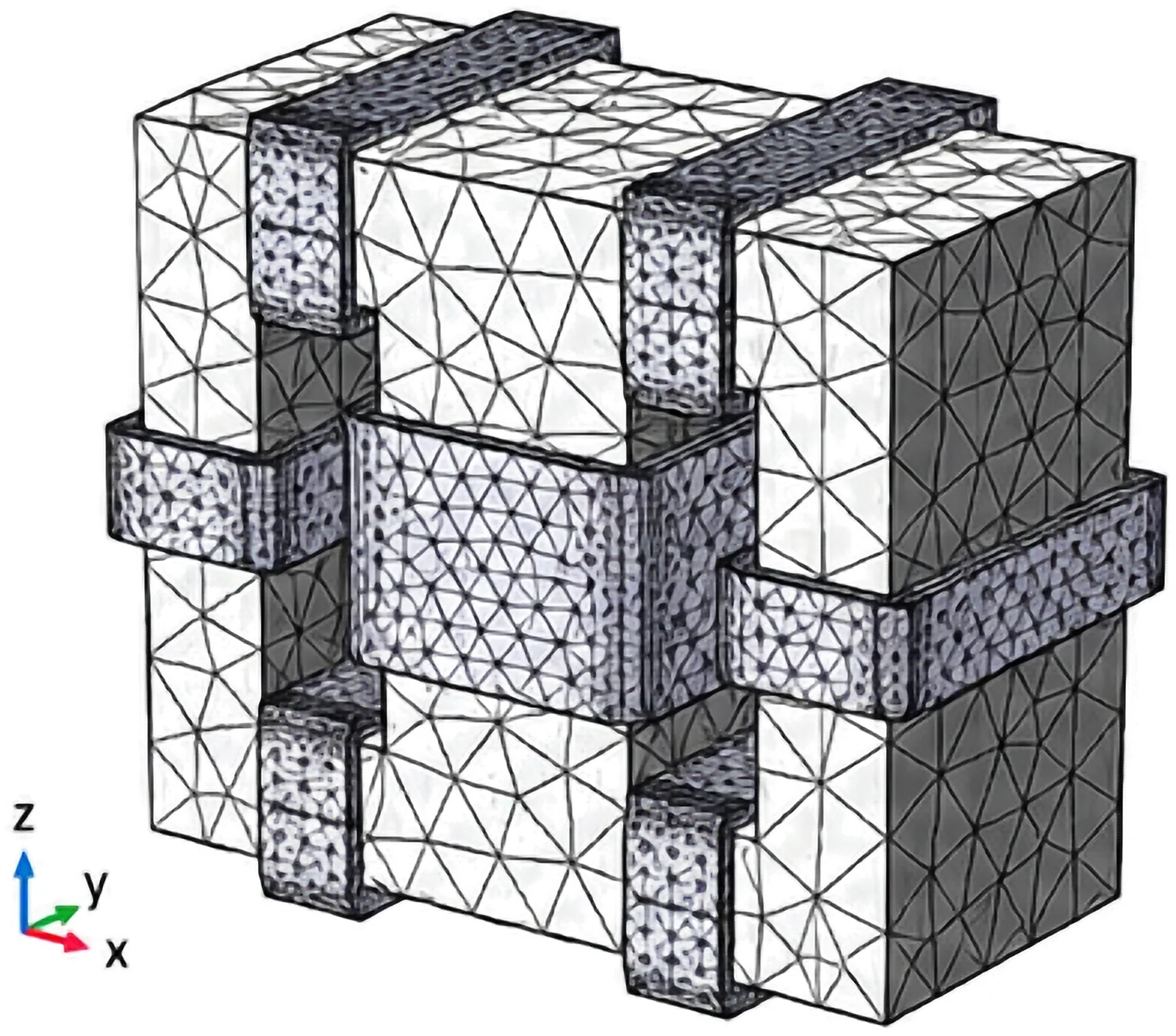
SICFCL Meshed 3D ﬁnite element model Geometry.

The proposed three-phase SICFCL is constructed using a commercial E-I magnetic core incorporating distributed three-phase AC windings around its outer limbs, while the DC excitation winding is installed on the central limb, as illustrated in Fig 4 For each phase, two AC windings are connected in series with the corresponding load phase. These windings are intentionally designed with unequal turn numbers of n,2n, and 3n. Accordingly, the total turns of each phase are arranged as (n+3n),(2n+2n*)*, and (3n+n), providing an identical total of *4n* turns per phase [29].

The selected turn ratios are intended to produce different impedance characteristics during fault conditions, thereby improving the current-limiting capability of the device. Nevertheless, Nevertheless, further investigation of different winding ratio configurations remains an area for future research.

Unlike the conventional single-phase SICFCL, the proposed three-phase configuration requires a lower DC magnetomotive force to establish core saturation. This is achieved because the magnetic flux is produced by the combined contribution of the three-phase AC MMF vectors, which generate a nonzero resultant owing to the intentionally unequal winding turn ratios. In addition, this winding arrangement promotes a more uniform magnetic flux distribution throughout the core, reducing localized magnetic losses. Consequently, the required number of turns in the DC winding, as well as the corresponding DC excitation current, is significantly lower than that of the conventional SICFCL design.

The implementation of unequal winding turn ratios is essential for establishing the impedance variation required during fault conditions. If identical turn numbers were assigned to all phase windings, the SICFCL would be incapable of effectively limiting balanced three-phase fault currents. Although the current-limiting ratio may vary between successive half cycles, its overall value remains consistent throughout a complete fault current cycle. Under equal-turn conditions, the fault currents in phases A, B, and C would have identical magnitudes, causing the corresponding ampere-turn contributions to cancel each other. As a result, the resultant fault-induced MMF would become nearly zero, preventing the core from producing the desired impedance change. Employing unequal turn ratios overcomes this limitation by generating a nonzero resultant MMF during fault conditions, thereby enabling effective fault current limitation. Moreover, the circuit-level description, detection logic, response time have been showed in [30]

## 3. Analytical method

### 3.1. Characteristics of overcurrent relay

Overcurrent protection systems for distribution networks, which are usually radial, are created using time-current characteristic curves that are given in (1), in addition to the operational characteristics of the OCRs, which are expressed [28,31] as follows:


$$\begin{array}{c} \int_{\text{0}}^{t}\left[\left((I_{fault}(t{)}^{\propto }/I_{P})-\text{1}\right)\right]dt=k\times TSM \end{array}$$ (1)

$$\begin{array}{c} t_{Primary}=[(A/(M_{Primary}^{P}-\text{1}))+B]\;X{TDS}_{Primary} \end{array}$$ (2)

$$\begin{array}{c} t_{Back-up}=[(A/(M_{Back-up}^{P}-\text{1}))+B]\;X{TDS}_{Back-up} \end{array}$$ (3)

$$\begin{array}{c} M_{Primary}=I_{Primary}/\;I_{Pick-upPrimary} \end{array}$$ (4)

$$\begin{array}{c} M_{Back-up}=I_{Back-up}/\;I_{Pick-upBack-up} \end{array}$$ (5)

where TSM and $I_{P}$Refer to the setting of the time and pick up the current of the relay, respectively; and $I_{Fault}(t)$refers to the short circuit current passing through the relay. Based on 𝐼𝐸𝐶60255 [39–43], 𝛼 and 𝑘 estimate the curve's shape and steepness, where the operating times of the primary and backup relays ($t_{Primary}$and $t_{Back-up}$) are determined based on their pickup currents ($I_{Pick-up}$) and Time Dial Settings (TDS), provided that the corresponding relay currents ($I_{Primary}$and $I_{Back-up}$) are known. The allowable range of TDS typically varies from 0.5 to 11, which defines the fastest and slowest operating times for a given current, respectively. The coefficients A, B, and p are constants used to define the overcurrent relay characteristic, representing inverse, very inverse, and extremely inverse types, respectively. In this study, the very inverse relay characteristic is used, with constant values of 3.922, 0.0982, and 2, respectively. To ensure proper coordination of overcurrent relays, the operating time of the backup relay must be greater than that of the primary relay for the same fault location by a Coordination Time Interval (CTI), and it varies due to the coordination status and desired tripping. A typical CTI value ranges between 0.2 and 0.5 seconds [32], As the primary protection device, it is crucial to make sure that the primary relay, which is closest to the fault, activates first. In order to isolate the fault, the backup relay activates after the designated Coordination Time Interval (CTI) if the primary relay is unable to trip. To attain appropriate coordination and reduce the possibility of relay miscoordination, this is essential. However, protection coordination becomes more difficult and necessitates in-depth analysis in networks with DG. This is because variations in fault levels can cause DG units to contribute to fault currents and alter the performance of protection devices Table 1.

**Table 1:** Description of symbols and abbreviations.

Symbol	Description
RE	Renewable energy
MG	Microgrid
PCC	Point of Common Coupling
DG	Distributed generators
CB	Circuit breaker
DFCL	Directional fault current limiter
MMF	Magnetomotive force
SICFCL	Saturated iron core fault current limiter
DC	Direct current
TSM	Time Setting Multiplier
CTI	Coordination Time Interval
OCR	Overcurrent Relay
$X_{d}^{\prime \prime }$	Sub-transient reactance
$X_{eq}$	Equivalent reactance
ETAP	Electrical Transient Analysis Program
EV	Electric vehicle
Z_MG_	Microgrid feeder impedance

### 3.2. Model configuration

**Fig 5:**
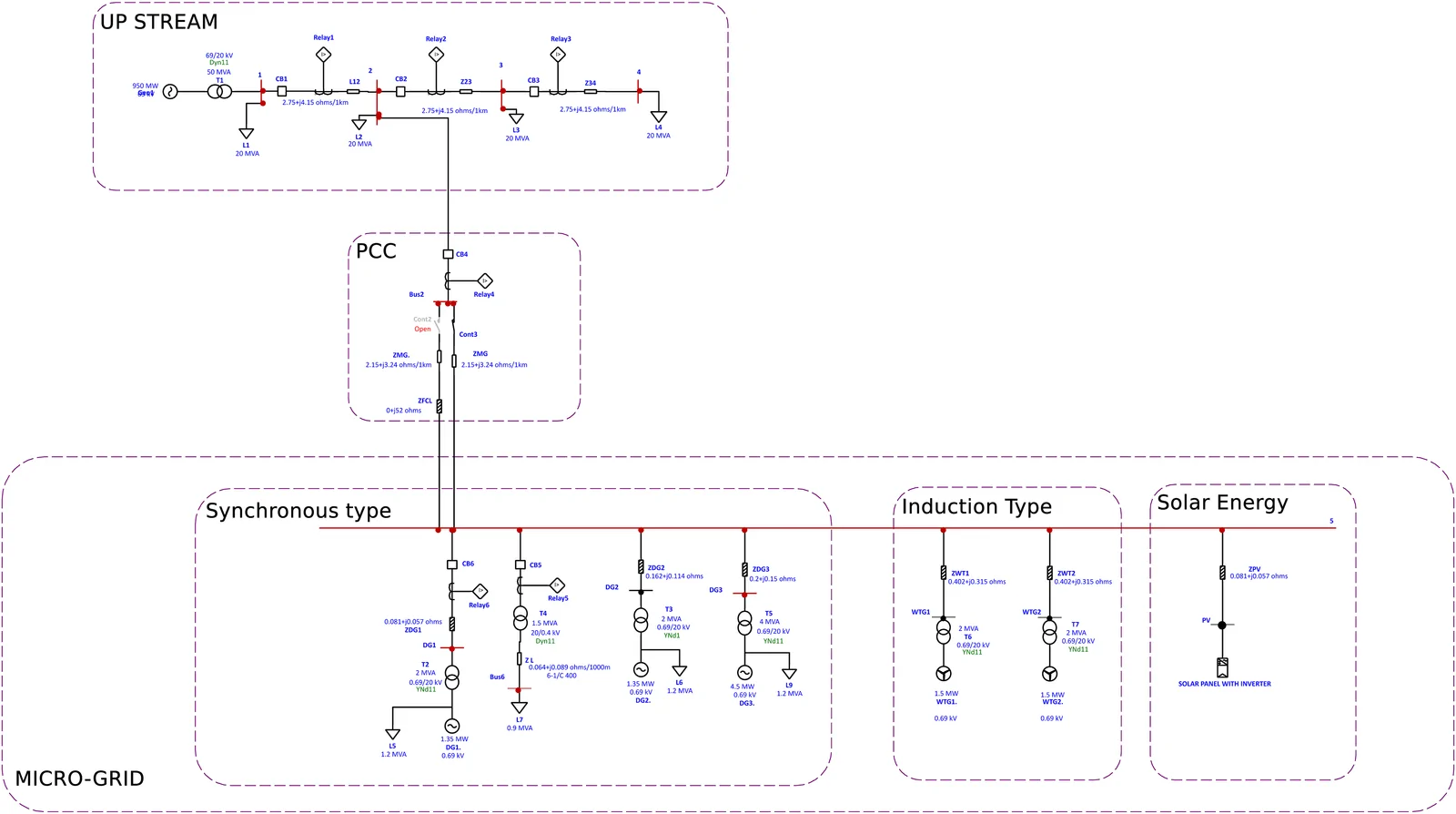
Single-line diagram of the study case.

Fig 5 shows the case study of a network that contains the upstream substation connected through a 50 MVA transformer to a transmission system with four OCRs, each of which follows a bus and a non-rotating load (LI to L4). The DFCL is connected at the PCC to the downstream network through bus 5. A synchronous generator representing DG l and a non-rotating load L5 are connected to bus DG l through a 2 MVA transformer. A non-rotating load L7 is connected to bus 5 through a 1.5 MVA transformer. A synchronous generator DG2 and a non-rotating load 1.6 MVA are added and connected to bus DG2 through a 2 MVA transformer. A synchronous generator DG3 and a non-rotating load 1.6 are added and connected to bus DG3 through a 4 MVA transformer. Two wing turbines with an induction generator (WTG1, WTG2) connected to bus DG4, DG5 through a 2 MVA transformer. PV system with an inverter connected to bus PV. Buses DGl, DG2, DG3, WTG1, WTG2, PV are connected to bus 5 through underground cables ZDGI, ZDG2, ZDG3, ZWT1, ZWT2,ZPV respectively, are subjected as shown. The whole system data and parameters are shown in Table 2.

**Table 2:** System data of upstream and downstream networks [15,28].

The network components	Data
Main substation	${\text{U}}_{\text{nQ}}=\text{69 KV},{\text{S}}_{\text{KQ}}^{\prime \prime }=\text{1000 MVA}$ ${\text{R}}_{\text{Q}}/{\text{Z}}_{\text{Q}}=\text{0}.\text{045}$
$Transformer(T_{\text{1}})$	${\text{S}}_{\text{rT}}=\text{50 MVA },{\text{u}}_{\text{kr}}=\text{20}.\text{5}\%$, ${\text{P}}_{\text{krT}}=\text{160 KW},{\text{T}}_{\text{r}}=\text{69}/\text{20KV}$
$Transformer(T_{\text{2}},T_{\text{3}},T_{\text{6}},T_{\text{7}})$	${\text{S}}_{\text{rT}}=\text{2}\text{ MVA },{\text{u}}_{\text{kr}}=\text{6}\%$, $u_{Rr}=\text{1}.\text{1}\%,T_{r}=\text{0}.\text{6}\text{9}/\text{2}\text{0}KV$
$Transformer(T_{\text{4}})$	${\text{S}}_{\text{rT}}=\text{1}.\text{5}\text{ MVA },{\text{u}}_{\text{kr}}=\text{6}.\text{5}\%$, ${\text{u}}_{\text{Rr}}=\text{1}\%,{\text{T}}_{\text{r}}=\text{20}/\text{0}.\text{4KV}$
$Transformer(T_{\text{5}})$	${\text{S}}_{\text{rT}}=\text{4}\text{ MVA },{\text{u}}_{\text{kr}}=\text{7}.\text{15}\%$, ${\text{u}}_{\text{Rr}}=\text{1}.\text{5}\%,{\text{T}}_{\text{r}}=\text{0}.\text{69}/\text{20KV}$
Overhead lines (${\text{Z}}_{\text{12}},{\text{Z}}_{\text{23}},\text{ and }{\text{ Z}}_{\text{34}})$	$\text{R}=\text{2}.\text{75 }\Omega \;,\text{X}=\text{3}.\text{24 }\Omega \;{\text{P}}_{\text{krT}}=\text{60 KW},{\text{T}}_{\text{r}}=\text{69}/\text{20KV}$
$\text{Overhead lines }\left({\text{Z}}_{\text{MG}}\right)$	R=2.15 Ω ,X=3.24 Ω
Underground cable$\left({\text{Z}}_{\text{DG1}}\right)$	R=2.15 Ω ,X=3.24 Ω
Underground cable$\left({\text{Z}}_{\text{DG2}}\right)$	R=0.162 Ω ,X=0.114 Ω
Underground cable$\left({\text{Z}}_{\text{DG3}}\right)$	R=0.2Ω ,X=0.15 Ω
Underground cable$\left({\text{Z}}_{\text{WT1}},{\text{Z}}_{\text{WT2}}\right)$	R=0.402 Ω ,X=0.315 Ω
Underground cable$\left({\text{Z}}_{\text{PV}}\right)$	R=0.081 Ω ,X=0.057 Ω
$\text{Non}-\text{rotating loads }\left({\text{L}}_{\text{1}}\text{ to }{\text{L}}_{\text{4}}\right)$	S=20 MVA,P.F=0.94
$\text{Non}-\text{rotating loads }\left({\text{L}}_{\text{7}}\right)$	S=0.9 MVA,P.F=0.97
$\text{N}-\text{rotating loads }\left({\text{L}}_{\text{5}},{\text{L}}_{\text{6}}\text{ and }{\text{L}}_{\text{8}}\right)$	S=1.2 MVA,P.F=0.95
$\text{Synchronous generator }\left({\text{DG}}_{\text{1}},{\text{DG}}_{\text{2}}\right)$	${\text{S}}_{\text{rG}}=\text{1}.\text{5}\text{ MVA },{\text{U}}_{\text{rG}}=\text{690 V}$, ${\text{X}}_{\text{d}}^{\prime \prime }=\text{18 },\text{P}.\text{F}=\text{0}.\text{9}\text{ lag}$
Induction generator (WTG1 , WTG2)	${\text{S}}_{\text{rG}}=\text{1}.\text{5}\text{ MW },{\text{U}}_{\text{rG}}=\text{690 V}$, ${\text{X}}_{\text{sc}}=\text{16}.\text{667 },\text{P}.\text{F}=\text{0}.\text{85 lag}$
Induction generator (WTG2)	${\text{S}}_{\text{rG}}=\text{1}.\text{5}\text{ MW },{\text{U}}_{\text{rG}}=\text{690 V}$, ${\text{X}}_{\text{sc}}=\text{17}.\text{33 },\text{P}.\text{F}=\text{0}.\text{85 lag}$
PV System with inverter	${\text{P}}_{\text{AC},\text{N}}=\text{1000 KW },{\text{I}}_{\text{AC},\text{N}}=\text{31}.\text{5}\text{ A}$,${\text{U}}_{\text{AC},\text{N}}=\text{20 KV}$

Miscoordination may manifest as some relays operating slowly or tripping needlessly. It is clear that the system's dependability deteriorates in these situations. Furthermore, in certain situations, particularly when synchronous-based DGs are assigned within MG, DG integration may cause the fault level to surpass the equipment's intended short-circuit capability, such as CB, that raise the possibility of their failure and damage, However, components of the protective system, like CBs, are more likely to fail to isolate the defective portion when one of the relays runs slowly as a result of miscoordination. CB hidden failure [33],is the term used to describe this stress-induced failure brought on by an increase in the strength and length of a fault current flowing through CB [29]. In such situations, inevitably the backup protection relays operation causes shutdown of a larger section. Thus, system reliability further decreases as more customers are affected. Therefore, to realize MG implementation and development with the purpose of improving reliability and resiliency of the grid, an effective SICFCL [30] should be proposed to address these issues.

### 3.3. Verification of the system

In order to determine the pick-up current of each overcurrent relay and the turns ratio of the current transformer feeding the relay, the ETAP is utilized in the short circuit study to calculate the current flowing through each feeder. In order to prevent picking up during overload, transient, and switching conditions, the pick-up current should be adjusted as low as feasible while yet being high enough. The maximum load current passing through each OCR determines its selection. The minimum and maximum pick-up current values for each relay are set at 1.2 and 2 times the maximum load current that passes through the relay during regular operation [25], The short-circuit analysis results presented in Table 3 are in a good agreement with those reported in [15], which further validates the developed model and confirms its suitability for conducting additional scenarios.

**Table 3:** Fault current results for the network connected to DG1.

Fault Location	Fault Current Contribution from Different Sources (KA)	
	Source Type	ETAP STUDY
**BUS 2**	SUBSTATION	1.88
	DG1	0.198
**BUS 3**	SUBSTATION	1.069
	DG1	0.112
**BUS 4**	SUBSTATION	0.723
	DG1	0.076
**BUS 5**	SUBSTATION	1.2
	DG1	0.21
**BUS 6**	SUBSTATION	0.408
	DG1	0.071
**BUS DG 1**	SUBSTATION	1.19
	DG1	0.21

**Fig 6:**
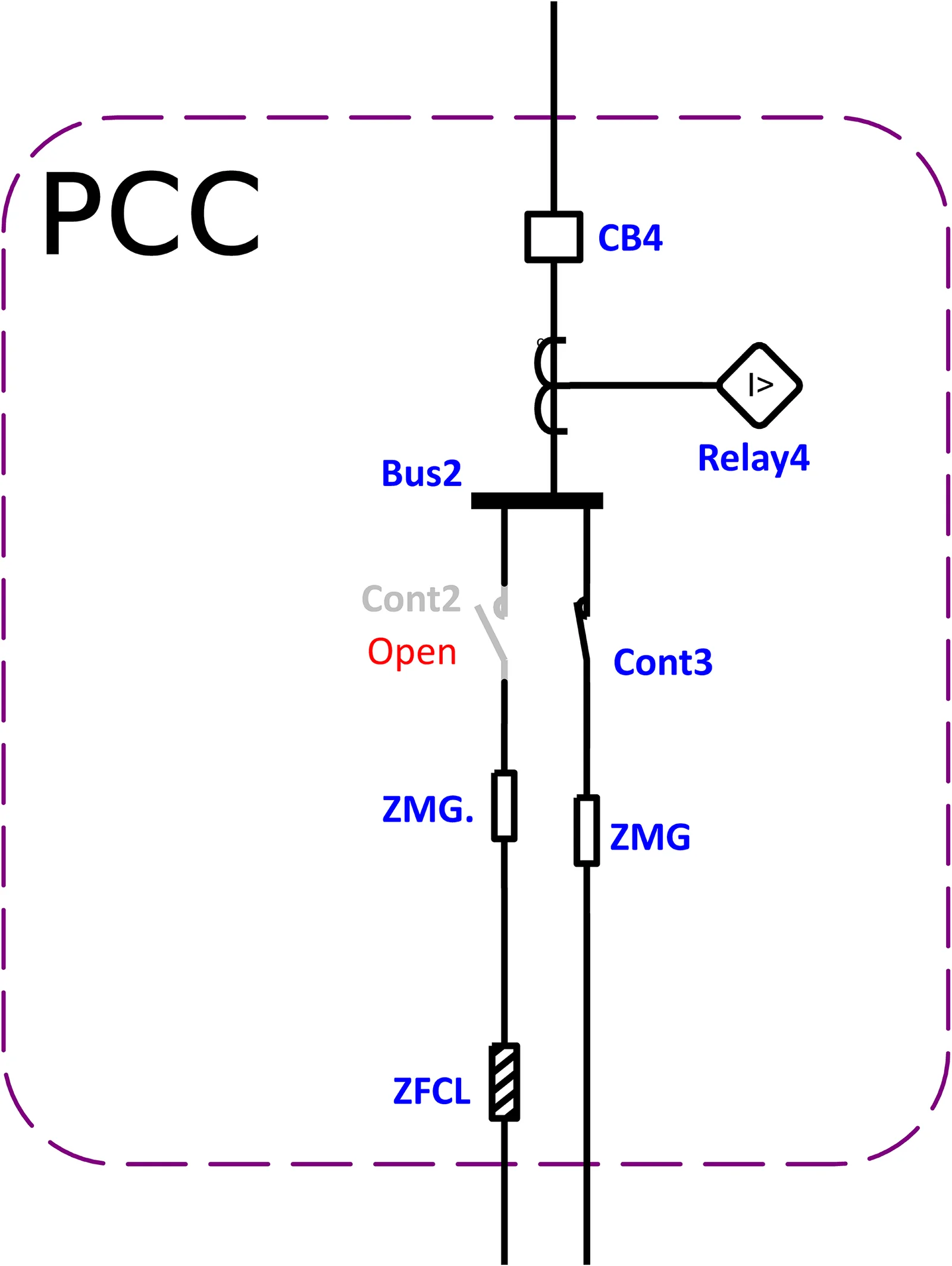
DFCL Configuration at the PCC.

One of the best ways to keep the protective coordination on utility distribution equipped with DGs is to use a fast-acting current limiting device, such as FCL, which can manage fault-current levels. Additionally, in areas where fault current magnitudes are anticipated to rise above the capacity of contemporary CBs, FCL considerably reduces power system stress. Therefore, some studies [34–37] have recommended alternative places to put FCL, such as in front of the point of integration in the DGs, bus tie, and feeders, The fault contribution of DGs or the feeder on which DG is integrated to the fault point will be managed by SICFCL [15] in this study. But it is important to mention that this method is not economical for an MG that might have multiple DGs. because by installing FCL inside the MG, the short circuit level of the MG during islanded mode is significantly reduced, rendering the overcurrent protection in the islanded MG inapplicable. This allows the FCL to be installed at PCC, which allows it to control the entire contribution from the various sources down in the MG as demonstrated in Fig 6.

#### 3.3.1. SICFCL Impedance control during fault conditions.

This paper's goal is to determine the suitable DFCL resistance value that limits the microgrid's contribution current by changing the impedance of the limiter in the fault condition, it can be implemented by adjusting the DC bias current dynamically to partially desaturate the core, or via tap-changer on the DC coil in advanced designs so we can get a various values to limit the desired current [30]. by doing this,miscoordination between overcurrent relays in distribution systems will be restored by achieving the appropriate relay coordination time intervals. micro grid configurations will change when DGs are in or out of service, which affects the fault current level in the fault at the upstream network.

## 4. Coordination analysis

OCR2 and OCR1 function as primary and backup relays, respectively, when a three-phase short circuit is applied at bus 3. Additionally, in the event of a three-phase fault on bus 4, OCR3 functions as the primary relay and overcurrent relays OCR2 as backup relays. When the OCR4 malfunctions, the relay OCR1 takes over as a backup relay. OCR5 and OCR4 function as primary and backup relays

when a three-phase short circuit is applied at bus 5. respectively. Also, when the fault occurs at bus DG1 OCR6 and OCR4 operate as primary an d back-up relays, to make this coordination we need to calculate the pickup current and the time delay for each relay by changing of Characteristics of overcurrent relays as shown in section 3.1 to make the CTI value ranges between 0.2 and 0.5 seconds as shown in Table 4 and 5.

**Table 4:** Setting values for each OCR for the base case with the DG1 integration.

Relay unit	Load Current (A)	C.T ratio	Pick-up current SETTING	TDS
**OCR 1**	749.1	1200:5	4.475	1.6
**OCR 2**	445.7	700:5	4.25	0.5
**OCR 3**	200.6	300:5	5	0.1
**OCR 4**	29.6	50:5	4.44	4.2
**OCR 5**	5.1	50:5	0.77	1.2
**OCR 6**	24.5	50:5	3.675	1.1

**Table 5:** Original relay coordination with the microgrid.

Fault location	Relay unit (primary, back-up)	Relay current (kA)	Operating time (s)	CTI (s)
**BUS 3**	OCR 2 (Primary)	1.181	0.321	0.208
	OCR 1 (Back-up)	1.075	0.529	
**BUS 4**	OCR 3 (Primary)	0.794	0.532	0.219
	OCR 2 (Back-up)	0.794	0.751	
**BUS 6**	OCR 5 (Primary)	0.474	0.189	0.327
	OCR 4 (Back-up)	0.409	0.516	
**BUS DG1**	OCR 6 (Primary)	1.19	0.106	0.278
	OCR 4 (Back-up)	1.19	0.384	

For faults in upstream network, the operation of the FCLs is desirable to limit fault current contribution of the MG and preserve the coordination of upstream over current relays ash shown in Table 5, but during a fault in downstream, current limitation by the FCL may decrease operational flexibility and reliability of the down- stream network. Loss of coordination between the upstream and downstream protective relays is one the of arisen problems, In order to overcome the problems resulting from fault occurrence downstream, a DFCL specially designed for microgrid is recommended.

The proposed DFCL presents a low resistance value in normal and downstream fault conditions and a high resistance value during the upstream fault conditions. In order to prevent miss-coordination between overcurrent relays in distribution systems, the goal of this research is to estimate the suitable value of DFCL Impedance that achieves the appropriate relay coordination time interval. Because DG units are connected to the distribution grid, this kind of miscoordination is to be expected.

### 4.1. Determination of MVA_sc_

Simplify fault analysis, complex power systems are commonly reduced using the Thevenin Equivalent technique. In this approach, the entire network upstream of the fault location is represented by an equivalent voltage source in series with an equivalent impedance. This simplification allows efficient calculation of the short-circuit current and, consequently, the MVAsc at the point of interest. Based on the Thevenin representation, the fault level can be expressed as a function of the system voltage and the equivalent impedance. A lower Thevenin impedance corresponds to a stronger system with higher fault current contribution, resulting in a higher MVAsc value. Conversely, higher impedance leads to reduced fault levels, which directly impacts the sensitivity and selectivity of protection schemes. The concept of MVAsc provides a convenient and voltage-independent measure of system strength, making it particularly useful in analyzing the impact of network modifications such as the integration of DG. As power systems become more complex, especially with the increasing penetration of renewable energy sources, accurate estimation of MVAsc using Thevenin-based models becomes essential for maintaining proper protection coordination and system stability, many formulas will be discussed as:


$$\begin{array}{c} {MVA}_{sc}=\text{3}\times V\times I_{sc} \end{array}$$ (6)

$$\begin{array}{c} {MVA}_{sc}=KV^{\text{2}}/Z_{th} \end{array}$$ (7)

For synchronous generators, the short-circuit contribution is governed by the sub-transient reactance, this parameter represents the generator’s internal impedance during the initial moments of a fault. Since $X_{d}^{\prime \prime }$ shown in (8) is typically small, generators contribute significantly to the fault current, especially in the first cycle.


$$\begin{array}{c} {MVA}_{sc}={MVA}_{rated}/X_{d}^{\prime \prime } \end{array}$$ (8)

as transformer impedance limits the flow of fault current between voltage levels. A lower per-unit or percentage impedance results in a higher MVAsc as shown in (9), indicating a stronger fault level. Transformers therefore play a critical role in controlling fault propagation across the network.


$$\begin{array}{c} {MVA}_{sc}={MVA}_{rated}/Z_{pu} \end{array}$$ (9)

As shown in (10), the transmission line and cables effect the fault current values longer feeders increase the total impedance, thereby reducing the fault level and limiting the short-circuit current at downstream buses, so it is very important to be taken into consideration.


$$\begin{array}{c} {MVA}_{sc}=KV^{\text{2}}/Z \end{array}$$ (10)

Unlike rotating generators, the short-circuit contribution of PV systems is limited by the inverter control. Therefore, the short-circuit capacity can be estimated using the inverter rated apparent power and its short-circuit current capability, where S_inverter_ is the inverter rated apparent power (MVA), and I_SC(inverter)_ is the inverter short-circuit current expressed in per unit or as a multiple of the rated current


$$\begin{array}{c} MVAsc(PV)=S_{inverter}\times I_{SC(inverter)} \end{array}$$ (11)

From the above formulations, it can be observed that MVAsc is strongly dependent on system impedance. Any modification in network configuration such as the integration of distributed generation or changes in feeder length, directly affects the equivalent impedance and, consequently, the fault level. This makes MVAsc a crucial parameter for evaluating protection coordination and system performance under fault conditions.

To validate the effectiveness of the proposed method under various network operating conditions, four representative simulation scenarios are considered from (A to E). In each scenario, the system fault level (MVAsc) is initially calculated to determine the available short-circuit capacity at the installation point. Subsequently, the required (Z_FCL_) is analytically derived based on the calculated fault level and the target fault current reduction. The simulation procedure and corresponding results for each scenario are discussed in the following subsections. All scenarios were tested based on the impact of the fault current limiter resistance comparing to not using it,as shown in Fig 6, at the PCC at Bus-2,a way to test the impact of FCL in ETAP to make the first parallel branch to the right through the contact 3 for the transmission line impedance to the Z_MG_ without the DFCL impedance,and the second branch through contactor 2,adding the FCL impedance with the same feeder impedance to the Z_MG_

For a three-phase fault at busbars 3, 4, 6 and DG1, the fault currents of the related OCRs are determined. The fault current contribution of main substation in upstream network Fig 5, is higher than that of distributed generator unit in downstream network irrespective of the fault location. This is because the rating of main substation is higher than that of DG unit as shown in Table 2.

**Fig 7:**
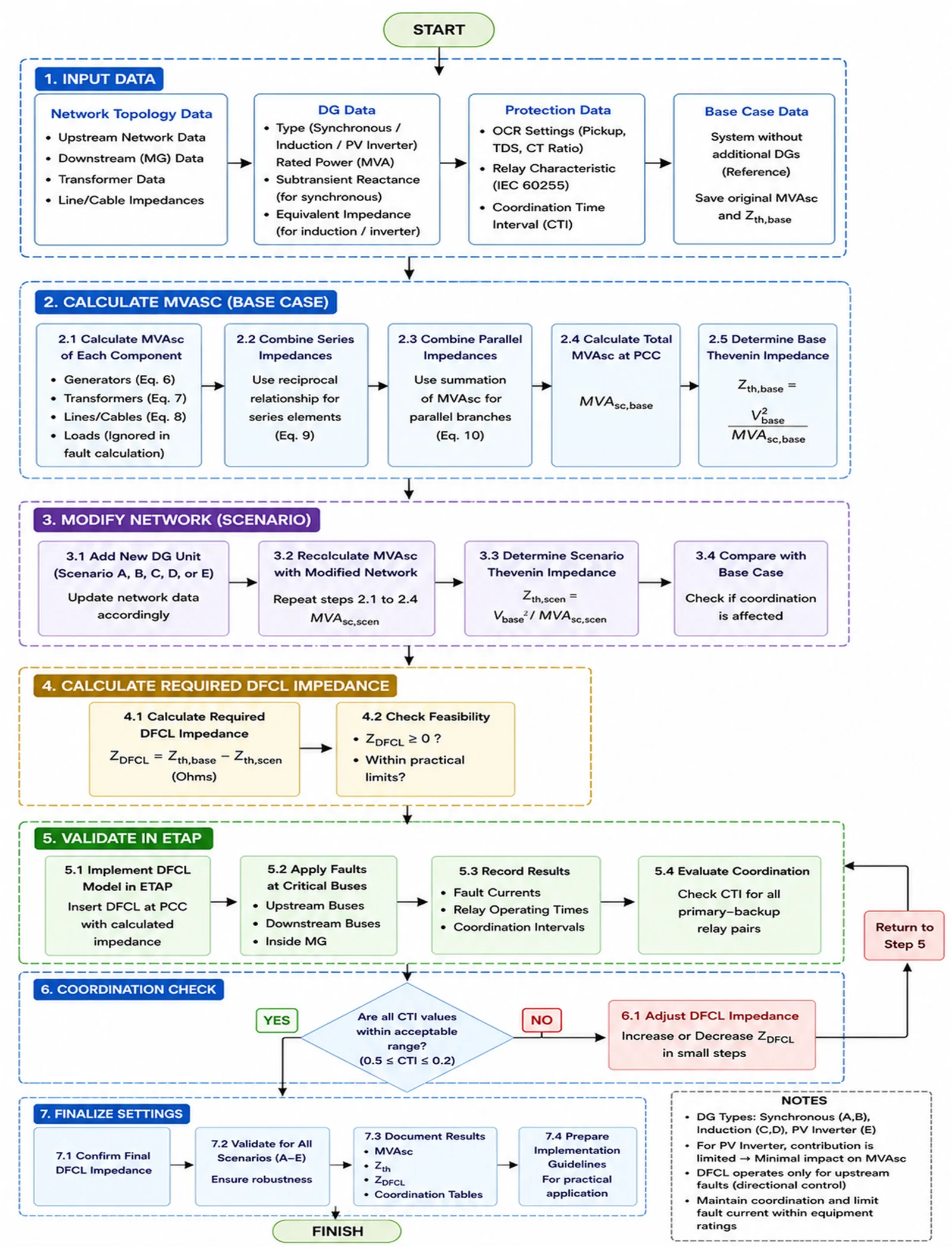
Flowchart of the Proposed DFCL Impedance Determination Methodology.

As shown in Fig 7 a detailed flowchart presents the sequential implementation steps, including input network data, MVAsc calculation, Thevenin equivalent impedance determination, DFCL impedance calculation, ETAP validation, and protection coordination assessment. This addition significantly improves the clarity and reproducibility of the proposed methodology.

## 5. Results and discussion

### 5.1. Original network configuration with only one DG

#### Proposed analytical procedure.

As shown in Fig 9, the original network configuration at the MG consists of a single synchronous generator (DG1), a step-up transformer (T2), the distributed generation feeder impedance (Z_DG1_), and the equivalent microgrid impedance (Z_MG_). To determine the equivalent Thevenin impedance seen from the fault location at Bus 2, which represents the PCC, and to evaluate the contribution of DGs to the fault current, the short-circuit capacity (MVA_sc_) of each component is first calculated individually using the system data provided in Table 1. These values are then combined according to subsection 4.1 and the corresponding series and parallel network connections, The calculation inputs and the corresponding MVAsc values are presented in Table 6.

**Table 6:** Input parameters and calculated short-circuit capacities for original configuration.

Component	Rating / Impedance	MVAsc
**DG1**	S = 1.5 MVA, Xd^” = 0.18.	8.33
**Transformer T2**	S = 2 MVA, Z = 6%.	33.33
**DG1 Feeder**	0.081 + j0.057	4038.55
**Microgrid Feeder**	2.15 + j3.24	102.868
**Equivalent Downstream combination**		6.65

The generation branch is connected in series with the equivalent microgrid impedance. Therefore, the equivalent short-circuit capacity is first obtained using also the reciprocal combination, after which it is converted into the corresponding Thevenin impedance shown in eq (7), so the DFCL impedance equals 64 ohm and it will be considered as the original Thevenin’s impedance.

### 5.2. Scenario A

#### A. Proposed analytical procedure.

As shown in Fig 10, integrating an additional 1.5 MVA synchronous generator, so the microgrid will contain DG1 + DG2, Following the methodology presented in original configuration, the short-circuit capacities of the modified system components are calculated using the same equations. The updated values and the resulting equivalent parameters are listed in Table 7.

**Table 7:** Input parameters and calculated short-circuit capacities for Scenario A.

Component	Rating / Impedance	MVAsc
**DG2**	S = 1.5 MVA, Xd” = 0.18.	8.33
**Transformer T3**	S = 2 MVA, Z = 6%.	33.33
**DG2 Feeder**	0.162 + j0.114	2019.28
**Microgrid Feeder**	2.15 + j3.24	102.868
**Equivalent Downstream combination**		**6.64**

**Fig 8:**
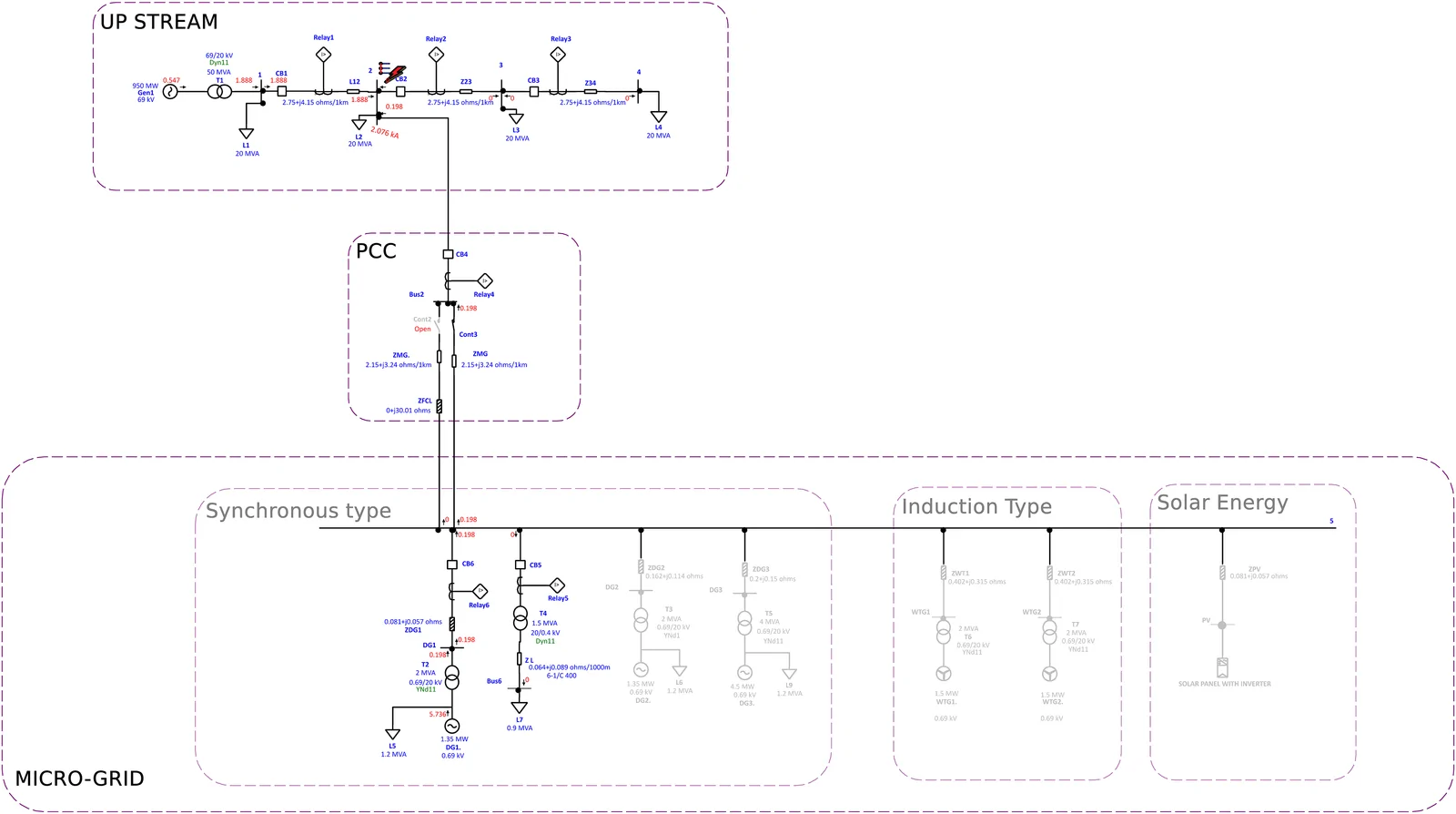
MG contribution with DG1 (original state).

Using the same methodology in original configuration, the new equivalent impedance is reduced to 33.99 Ω due to the DG integration, so by inserting a DFCL with an impedance of 30.01 Ω, the system equivalent Thevenin impedance is restored to its original value before DG integration. Consequently, the fault current almost returns to the base value as shown in Fig 10.

#### B. DFCL impedance validation.

Before adding DG2, the contribution of the microgrid as shown in Fig 8 with only DG1 was about 0.198 kA, the coordination time values and parameters were adjusted according to this case for the entire up and down network as shown in Table 8. Due to adding a new generation source this will affect the fault current by increasing its value because the contribution from the microgrid will be increased.

**Table 8:** Fault current results for the network connected to DG1&DG2.

Fault Location	Relay unit (primary, back-up)	Relay current (kA)	Operating time (s)	CTI (s)
BUS 3	OCR 2 (Primary)	1.233	0.302	0.898
	OCR 1 (Back-up)	1.041	1.2	
BUS 4	OCR 3 (Primary)	0.818	0.509	0.175
	OCR 2 (Back-up)	0.818	0.684	
BUS 6	OCR 5 (Primary)	0.493	0.189	0.328
	OCR 4 (Back-up)	0.376	0.547	
BUS DG1	OCR 6 (Primary)	1.367	0.105	0.279
	OCR 4 (Back-up)	1.188	0.384	

**Fig 9:**
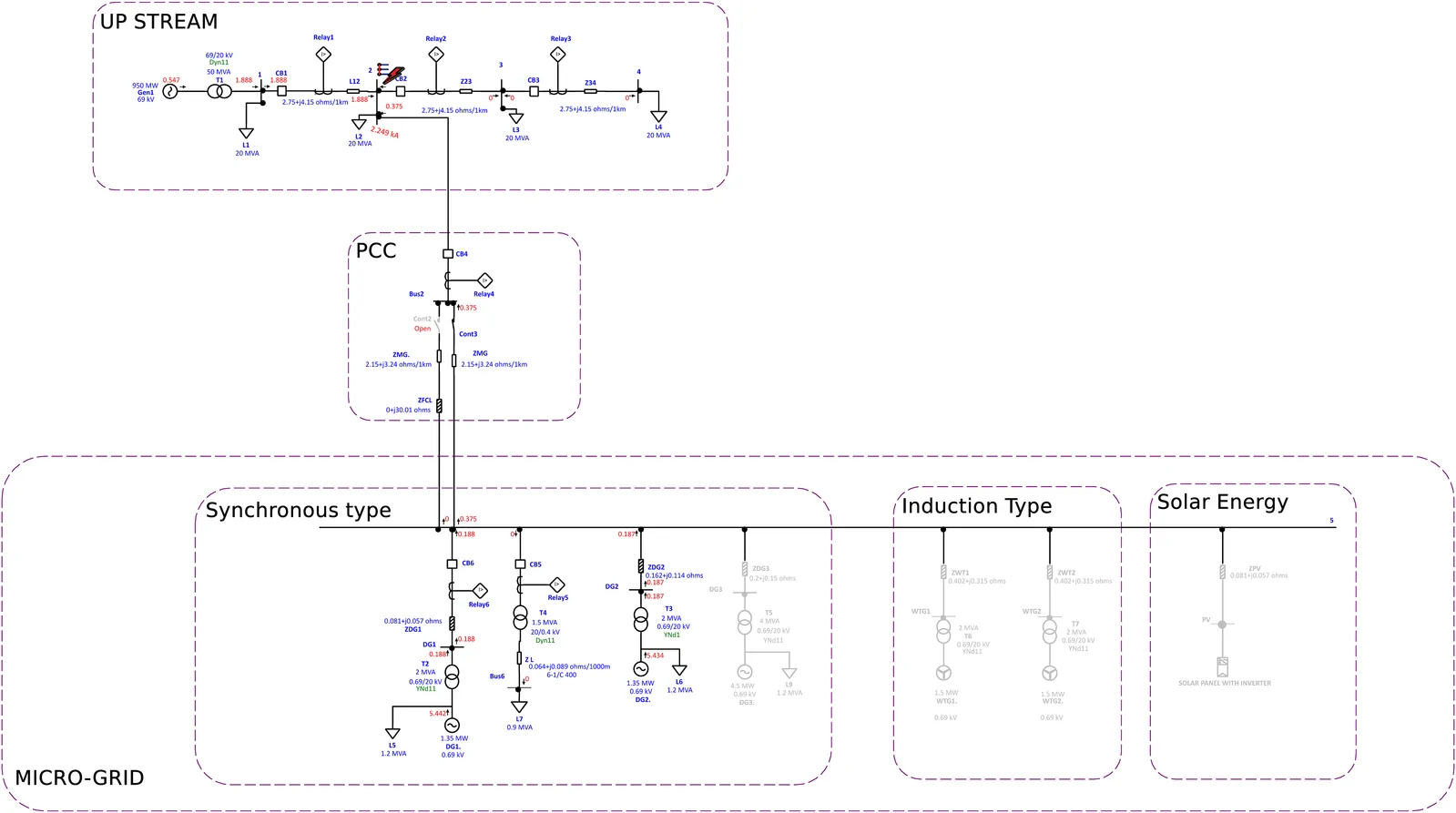
MG contribution after adding DG2.

As shown in Table 8 and Fig 9, integrating an additional 1.5 MVA synchronous generator (DG2) increases the DG contribution to 0.375 kA, resulting in a total fault current of 2.249 kA, this leads to miscoordination at BUS-3 and BUS-4 because those buses are located at the upstream network which the contribution current will flow through.

**Fig 10:**
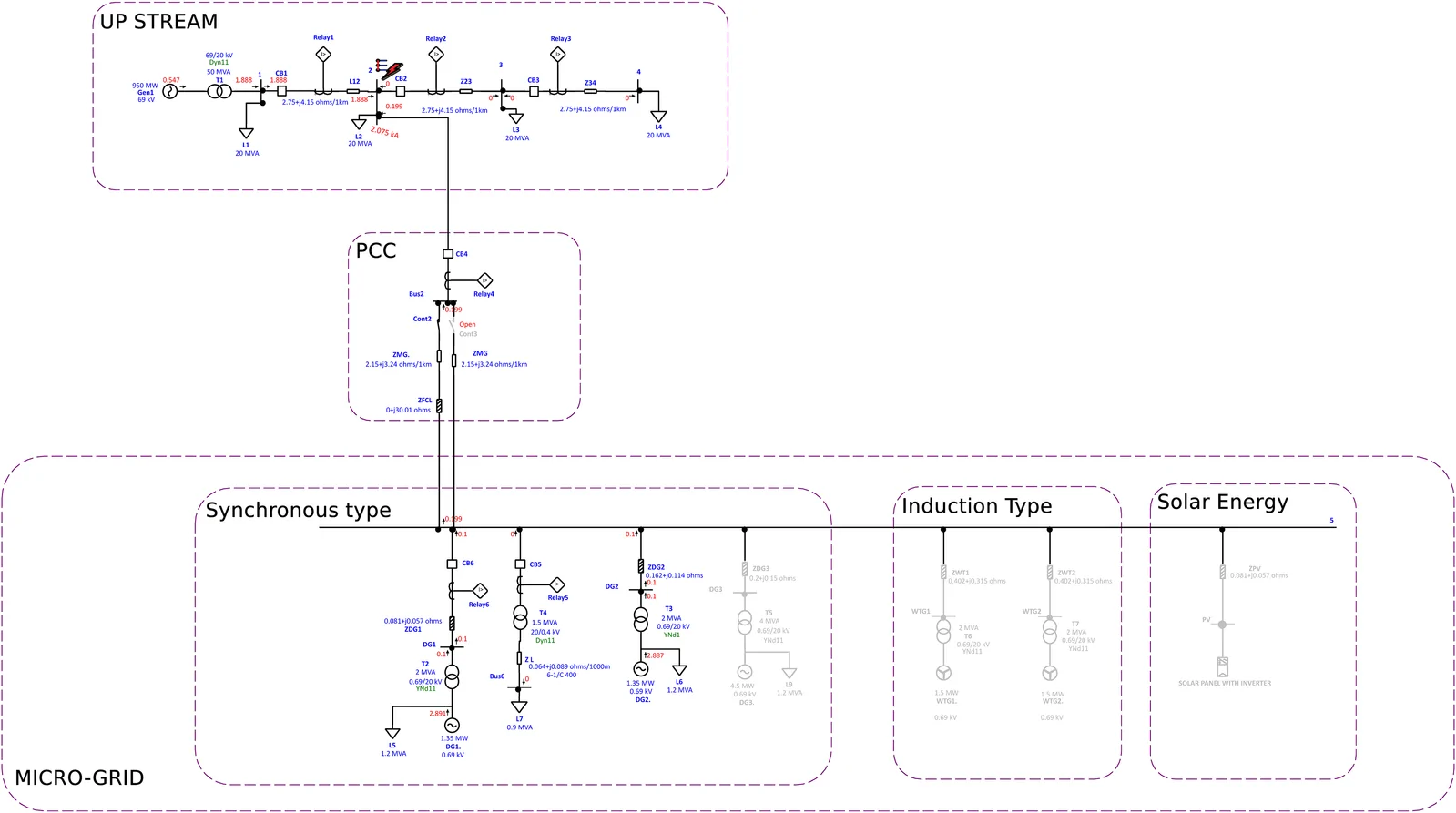
Restoration of the system parameters by Implement DFCL at PCC.

The newly added DG contributes 7.87% of the total fault current. Accordingly, a DFCL with a calculated impedance of 30.01 Ω is required only for upstream faults, where it limits the microgrid fault-current contribution to the utility grid. For downstream faults as shown in Fig 10, the DFCL is intentionally blocked since the microgrid protection coordination has already been established without current limitation, and the upstream utility contribution remains constant. Operating the DFCL under downstream fault conditions would unnecessarily reduce the fault current, potentially leading to protection miscoordination. Hence, directional control is indispensable to ensure that the DFCL is activated exclusively during upstream faults, as verified in Tables 9 and Table 10.

**Table 9:** Relay coordination after adding None-DFCL.

Fault Location	Relay unit (primary, back-up)	Relay current (kA)	Operating time (s)	CTI (s)
**BUS 3**	OCR 2 (Primary)	1.181	0.321	0.208
	OCR 1 (Back-up)	1.076	0.529	
**BUS 4**	OCR 3 (Primary)	0.794	0.533	0.218
	OCR 2 (Back-up)	0.794	0.751	
**BUS 6**	OCR 5 (Primary)	0.354	0.189	1.382
	OCR 4 (Back-up)	0.157	1.571	
**BUS DG1**	OCR 6 (Primary)	0.512	0.123	0.504
	OCR 4 (Back-up)	0.318	0.627	

**Table 10:** Relay coordination after adding DFCL.

Fault Location	Relay unit (Primary,Back-up)	Relay current (kA)	Operating time (s)	CTI (s)
**BUS 3**	OCR 2 (Primary)	1.181	0.321	0.208
	OCR 1 (Back-up)	1.076	0.529	
**BUS 4**	OCR 3 (Primary)	0.794	0.533	0.218
	OCR 2 (Back-up)	0.794	0.751	
**BUS 6**	OCR 5 (Primary)	0.493	0.189	0.328
	OCR 4 (Back-up)	0.376	0.517	
**BUS DG1**	OCR 6 (Primary)	1.367	0.105	0.279
	OCR 4 (Back-up)	1.188	0.384	

### 5.3 Scenario B

#### A. Proposed analytical procedure.

**Fig 11:**
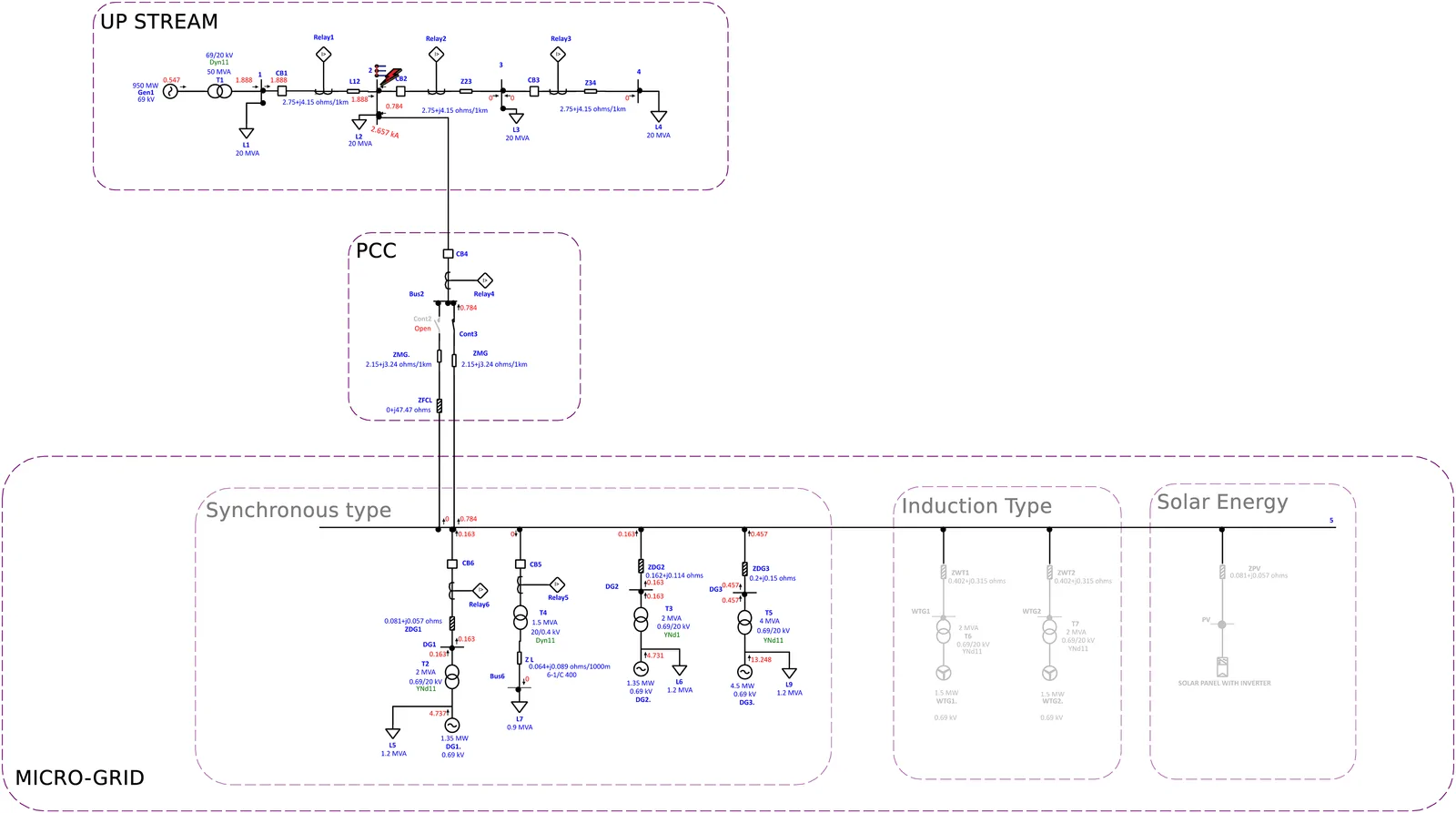
MG contribution after adding DG3.

As shown in Fig 11,adding a larger 5 MVA synchronous generator,so the microgrid will contain (DG1 + DG2 + DG3), Following the methodology presented in original configuration, the short-circuit capacities of the modified system components are calculated using the same equations. The updated values and the resulting equivalent parameters are listed in Table 11.

**Table 11:** Input parameters and calculated short-circuit capacities for Scenario B.

Component	Rating / Impedance	MVAsc
**DG3**	S = 5 MVA, Xd” = 0.18.	27.78
**Transformer T5**	S = 4 MVA, Z = 7.15%.	55.94
**DG3 Feeder**	0.2 + j0.15	1600
**Microgrid Feeder**	2.15 + j3.24	102.868
**Equivalent Downstream combination**		**6.64**

**Fig 12:**
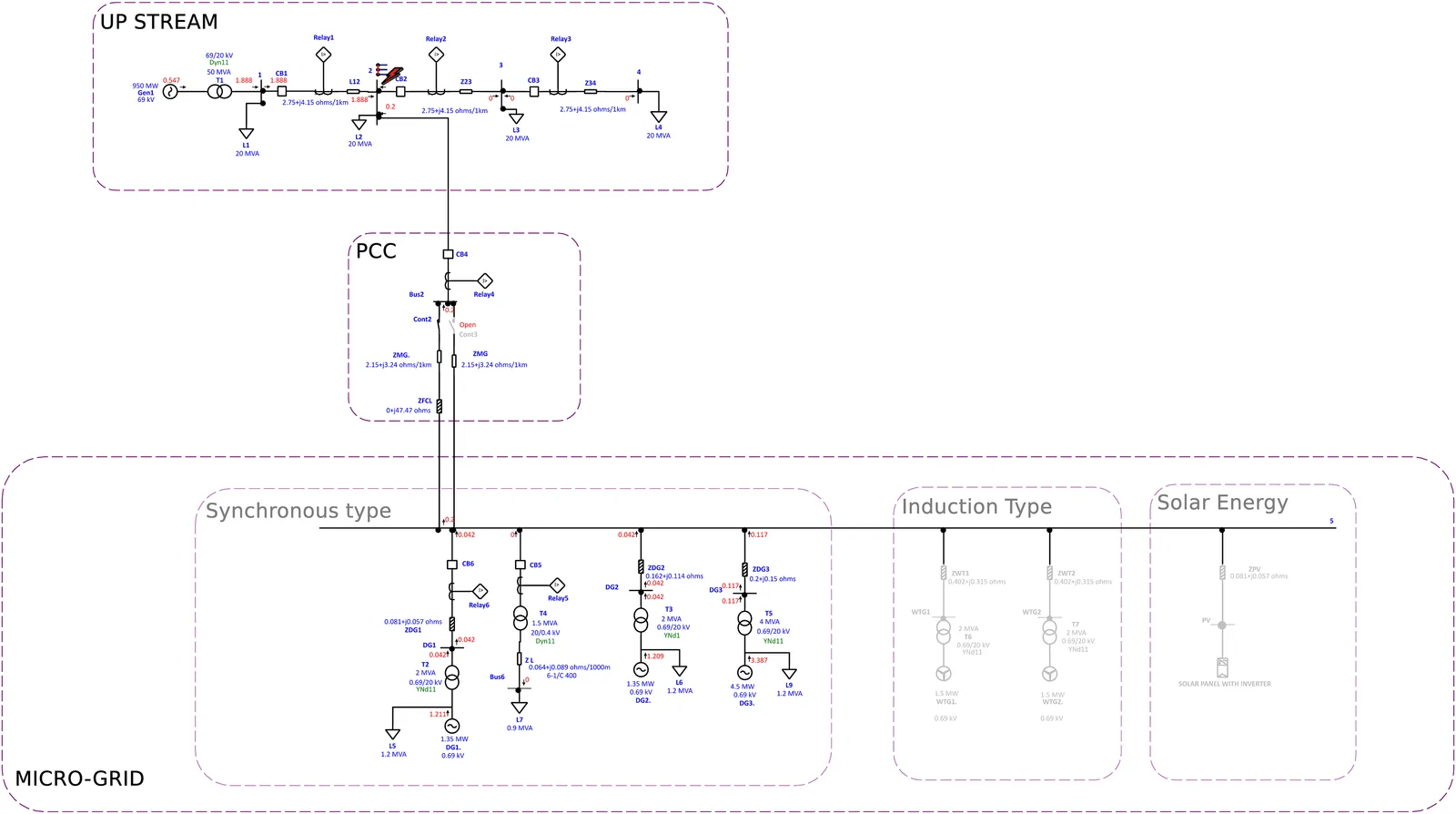
Restoration of the system parameters by Implement DFCL at PCC.

Using the same methodology in original configuration, the new equivalent impedance is reduced to 16.53 Ω due to the DG integration. By inserting a DFCL with an impedance of 47.47 Ω, the system equivalent Thevenin impedance is restored to its original value before DG integration. Consequently, the fault current almost returns to the base value as shown in Fig 12.

#### B. DFCL Impedance Validation.

Adding a larger 5 MVA synchronous generator (DG3), as shown in Fig 7 and Table 12, the contribution current turned from 0.375 KA to 0.784 KA resulting in a total fault current of 2.657 kA

**Table 12:** Relay coordination after adding DG 3.

Fault Location	Relay unit (primary, back-up)	Relay current (kA)	Operating time (s)	CTI (s)
**BUS 3**	OCR 2 (Primary)	1.352	0.269	0.256
	OCR 1 (Back-up)	0.96	0.525	
**BUS 4**	OCR 3 (Primary)	0.868	0.463	0.113
	OCR 2 (Back-up)	0.868	0.576	
**BUS 6**	OCR 5 (Primary)	0.539	0.189	0.474
	OCR 4 (Back-up)	0.3	0.663	
**BUS DG1**	OCR 6 (Primary)	1.932	0.105	0.279
	OCR 4 (Back-up)	1.185	0.384	

As shown in Table 8 Bus-4 and Bus-3 which are the upstream buses will be affected comparing to the original values at Table 5, but the coordination of the downstream microgrid will be accepted, by Following the detailed analysis presented for Scenario A, the same MVAsc-based methodology is applied to this one to get the suitable impedance for the DFCL shown in Fig 8, we will be able to restore the fault current as the base value

Following the detailed analysis presented for Scenario B, with a calculated impedance of 47.47 Ω as shown in Fig 12, we will be able to restore the fault current almost to the base value

### 5.4 Scenario C

#### A. Proposed analytical procedure.

**Fig 13:**
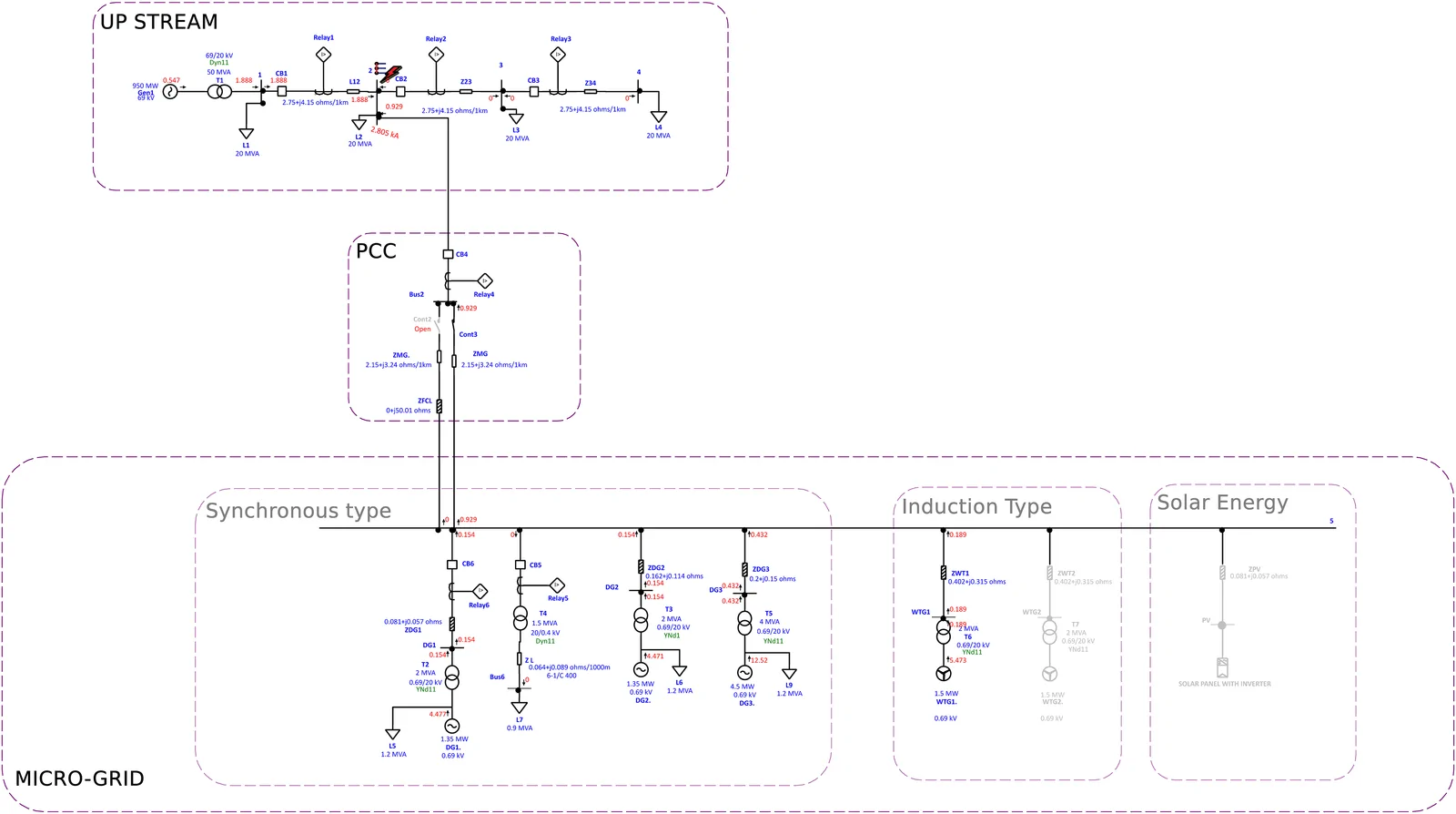
MG contribution after adding WT1.

As shown in Fig 13, integrating the 1.765 MVA induction generator, so the microgrid will contain (DG1 + DG2 + DG3 + WT1), Following the methodology presented in original configuration, the short-circuit capacities of the modified system components are calculated using the same equations. The updated values and the resulting equivalent parameters are listed in Table 13.

**Table 13:** Input parameters and calculated short-circuit capacities for Scenario C.

Component	Rating / Impedance	MVAsc
**WT 1**	S = 1.765 MVA, Xeq = 0.16667.	10.59
**Transformer T6**	S = 2 MVA, Z = 6%.	33.33
**WT 1 Feeder**	0.4 + j0.315	785.64
**Microgrid Feeder**	2.15 + j3.24	102.868
**Equivalent Downstream combination**		**7.96**

**Fig 14:**
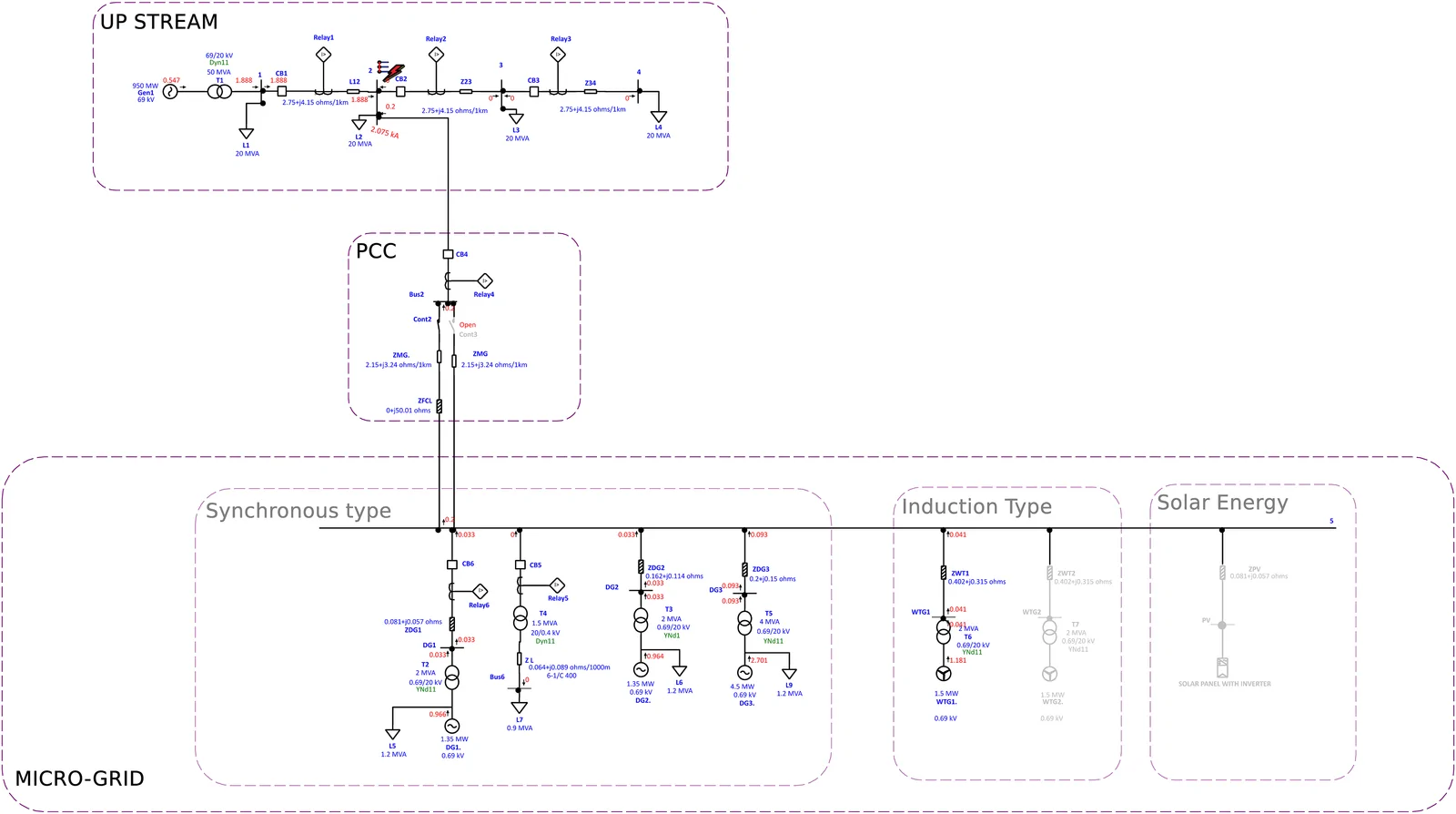
Restoration of the system parameters by Implement DFCL at PCC.

Using the same methodology in original configuration, the new equivalent impedance is reduced to 13.99 Ω due to the DG integration. By inserting a DFCL with an impedance of 50.01 Ω, the system equivalent Thevenin impedance is restored to its original value before DG integration. Consequently, the fault current almost returns to the base value as shown in Fig 14.

#### B. DFCL impedance validation.

Integrating a 1.765 MVA induction generator (WT1) connected to bus-5 through step up transformer, this will lead to increase the contribution of the microgrid so this will lead to a miscoordination and increase the DG contribution to 0.929 kA, as shown in Fig 13 and Table 14 producing a total fault current of 2.805 kA

**Table 14:** Relay coordination after adding WT1.

Fault Location	Relay unit (primary, back-up)	Relay current (kA)	Operating time (s)	CTI (s)
**BUS 3**	OCR 2 (Primary)	1.388	0.261	1.156
	OCR 1 (Back-up)	0.935	1.42	
**BUS 4**	OCR 3 (Primary)	0.883	0.451	0.1
	OCR 2 (Back-up)	0.883	0.552	
**BUS 6**	OCR 5 (Primary)	0.554	0.189	0.461
	OCR 4 (Back-up)	0.276	0.65	
**BUS DG1**	OCR 6 (Primary)	2.178	0.105	0.279
	OCR 4 (Back-up)	1.183	0.384	

As shown in Table 14,Bus-4 and Bus-3 which are the upstream buses will be affected comparing to the original values at Table 5, but the coordination of the downstream microgrid will be accepted, by Following the detailed analysis presented for Scenario C, with a calculated impedance of 50.01 Ω as shown in Fig 14, we will be able to restore the fault current almost to the base value

### 5.5. Scenario D

#### A. Proposed analytical procedure.

**Fig 15:**
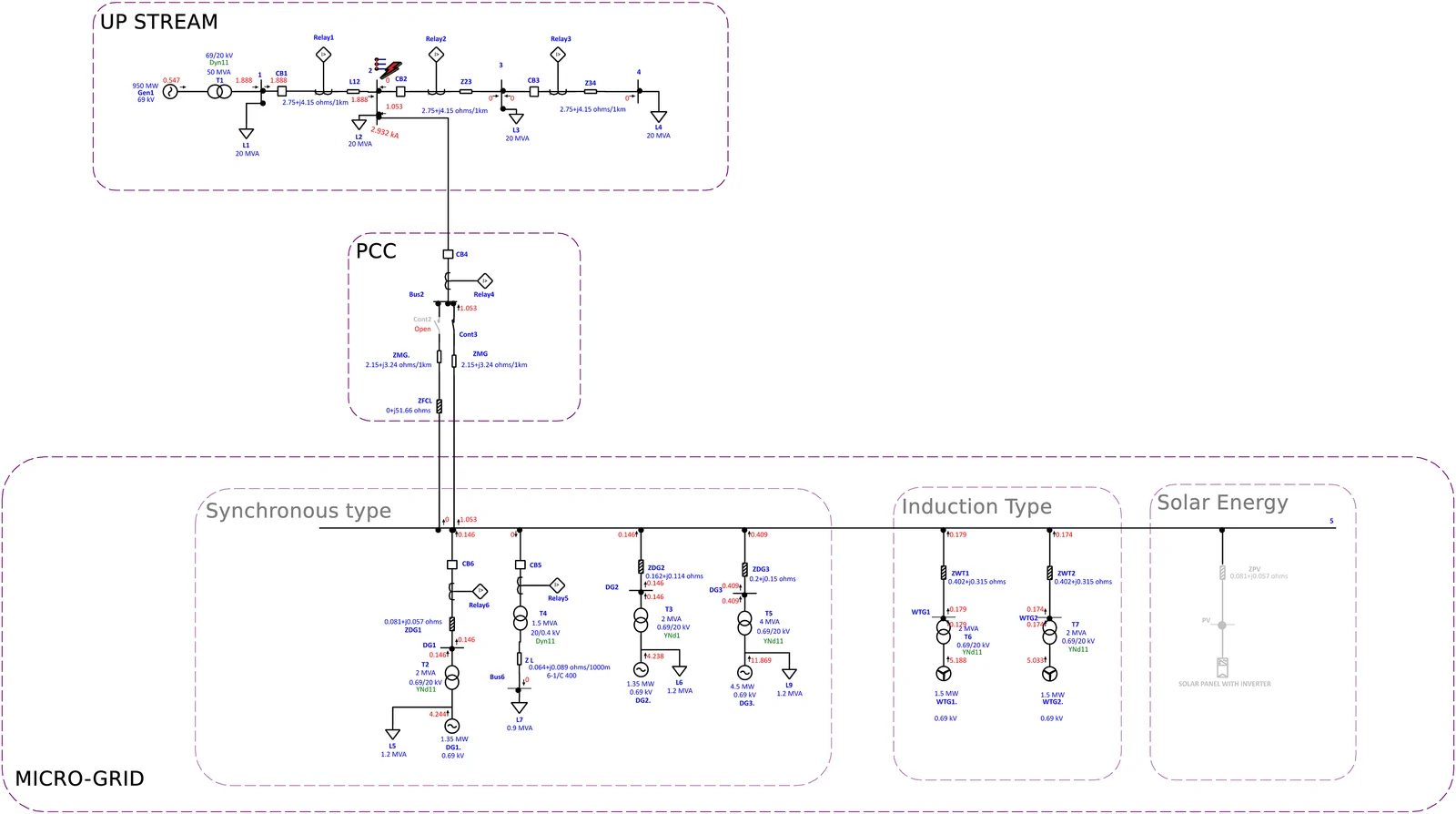
MG contribution after adding WT2.

As shown in Fig 15, a second induction generator, with a higher internal impedance is integrated, so the microgrid will contain (DG1 + DG2 + DG3 + WT1 + WT2), Following the methodology presented in original configuration, the short-circuit capacities of the modified system components are calculated using the same equations. The updated values and the resulting equivalent parameters are listed in Table 15.

**Table 15:** Input parameters and calculated short-circuit capacities for Scenario D.

Component	Rating / Impedance	MVAsc
**WT 2**	S = 1.765 MVA, Xeq = 0.1733.	10.185
**Transformer T7**	S = 2 MVA, Z = 6%.	33.33
**WT 2 Feeder**	0.4 + j 0.315	785.64
**Microgrid Feeder**	2.15 + j 3.24	102.868
**Equivalent Downstream combination**		7.72

**Fig 16:**
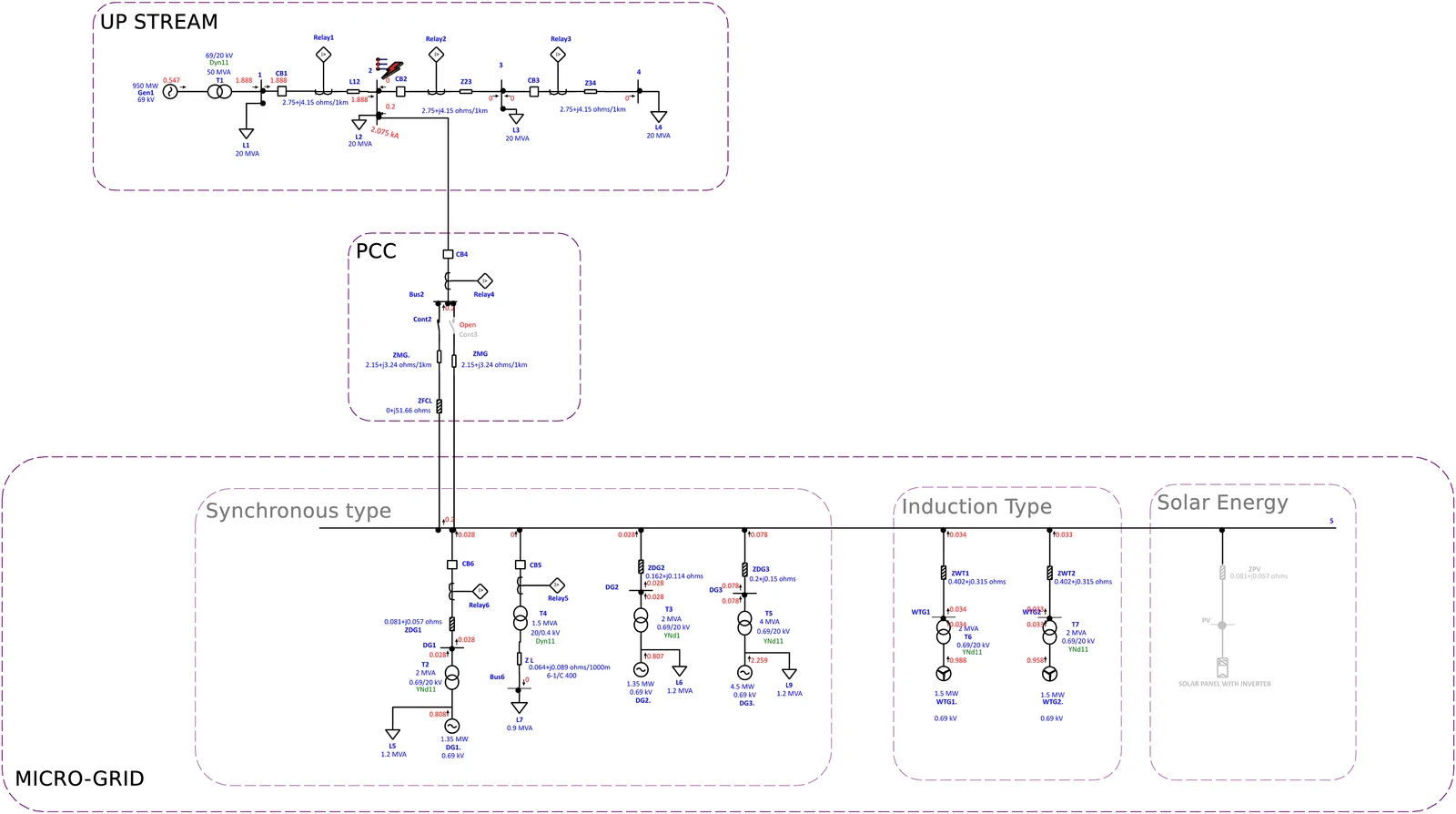
Restoration of the system parameters by Implement DFCL at PCC.

Using the same methodology in original configuration, the new equivalent impedance is reduced to 12.34 Ω due to the DG integration. By inserting a DFCL with an impedance of 51.66 Ω, the system equivalent Thevenin impedance is restored to its original value before DG integration. Consequently, the fault current almost returns to the base value as shown in Fig 16.

#### B. DFCL impedance validation.

Second induction generator (WT2) with a higher internal impedance is integrated. As shown in Fig 15 the overall DG contribution increases to 1.053 kA and the total fault current reaches 2.932 kA,.

As shown in Table 16,Bus-4 and Bus-3 which are the upstream buses will be affected comparing to the original values at Table 4, but the coordination of the downstream microgrid will be accepted, by with a Calculated DFCL impedance of 51.66 Ω shown in Fig 16, we will be able to restore the fault currents almost to the base value.

**Table 16:** Relay coordination after adding WT2.

Fault Location	Relay unit (primary, back-up)	Relay current (kA)	Operating time (s)	CTI (s)
**BUS 3**	OCR 2 (Primary)	1.388	0.261	1.156
	OCR 1 (Back-up)	0.935	1.42	
**BUS 4**	OCR 3 (Primary)	0.883	0.451	0.1
	OCR 2 (Back-up)	0.883	0.552	
**BUS 6**	OCR 5 (Primary)	0.554	0.189	0.461
	OCR 4 (Back-up)	0.276	0.65	
**BUS DG1**	OCR 6 (Primary)	2.178	0.105	0.279
	OCR 4 (Back-up)	1.183	0.384	

### 5.6. Scenario E

#### Proposed analytical procedure.

**Fig 17:**
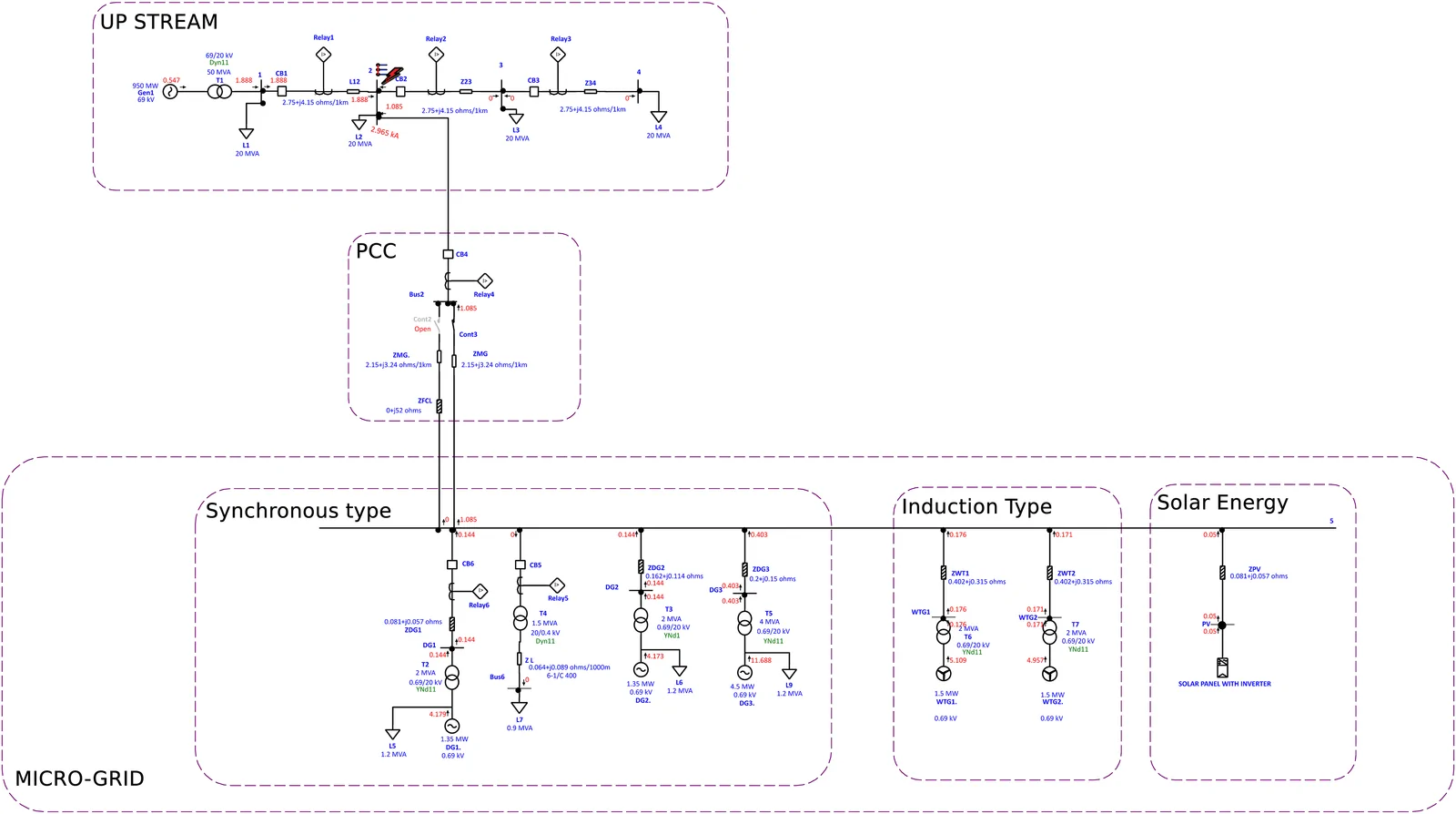
MG contribution after adding PV.

As shown in Fig 17, 1 MVA inverter-based PV system is added. The total fault current increases slightly to 2.965 kA, while the overall DG contribution reaches 1.085 kA,the integration of solar energy systems interfaced through power electronic converters introduces a fundamentally different behavior compared to conventional rotating generators during fault conditions. Unlike synchronous or induction machines, inverter-based DGs As Solar Panels are inherently controlled and typically designed with current-limiting control strategies that restrict their output current to a value close to the rated current, even under short-circuit conditions. As a result, their fault current contribution is considerably lower than that of conventional generators, which are capable of delivering only several times their rated current during the sub-transient period. Moreover, Several studies in the literature have confirmed that photovoltaic (PV) systems contribute limited fault current, typically in the range of 1.5 to 2 times of their rated current [38], depending on the inverter control scheme. In our study we used to get the worst case which consider to be twice the rated current to study the impact of the PV integration

- For PV inverter

Unlike synchronous and induction generators, inverter-based photovoltaic (PV) systems have a limited fault current contribution due to the current-limiting capability of the power electronic converter as shown in Fig 17, the total fault current increases slightly to 2.965 kA. Without causing a miscoordination if we used the DFCL impedance in previous scenario due to its small effect will, the short-circuit capacity of a PV unit is commonly estimated based on the inverter apparent power rating and its maximum short-circuit current capability. Accordingly, the short-circuit capacity of the PV inverter can be calculated using eq (11) is equal to 2 MVA Table 17.

**Table 17:** Relay coordination after adding PV.

Fault Location	Relay unit (primary, back-up)	Relay current (kA)	Operating time (s)	CTI (s)
**BUS 3**	OCR 2 (Primary)	1.183	0.320	0.209
	OCR 1 (Back-up)	1.074	0.529	
**BUS 4**	OCR 3 (Primary)	0.795	0.531	0.217
	OCR 2 (Back-up)	0.795	0.748	
**BUS 6**	OCR 5 (Primary)	0.510	0.189	0.461
	OCR 4 (Back-up)	0.059	0.65	
**BUS DG1**	OCR 6 (Primary)	1.529	0.105	0.279
	OCR 4 (Back-up)	0.206	0.384	

**Fig 18:**
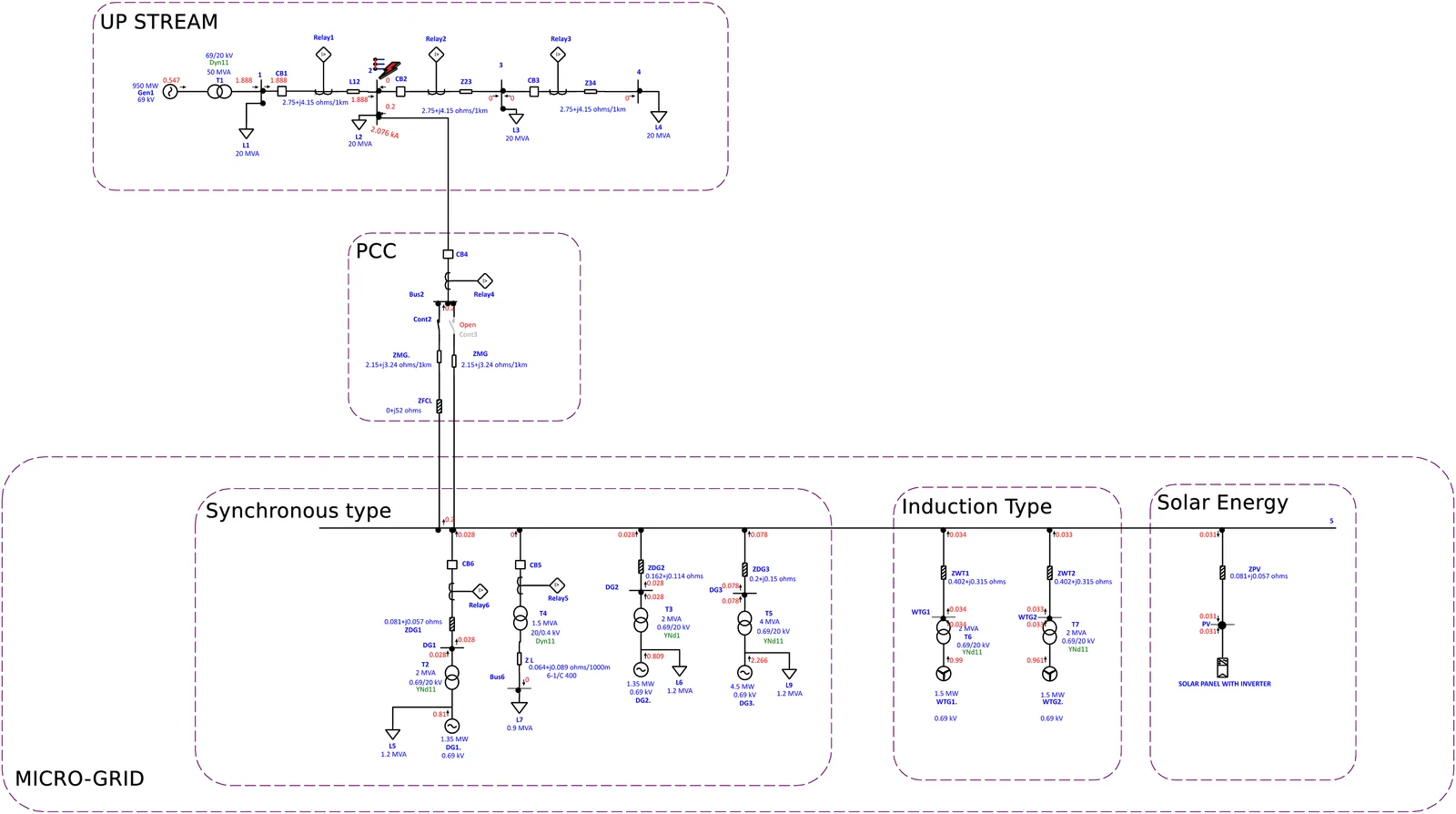
Restoration of the system parameters by implementing DFCL at PCC.

Their equivalent short-circuit capacity is calculated using the reciprocal relationship, so the total series MVAsc connected for the new branch is equal to 7.72 MVA. The generation branch is connected in series with the equivalent microgrid impedance. Therefore, the equivalent short-circuit capacity is first obtained using also the reciprocal combination with previous cases, after which it is converted into the corresponding Thevenin impedance shown in eq (7), the new equivalent impedance is reduced to 12 Ω due to the DG integration. By inserting a DFCL with an impedance of 52 Ω, the system equivalent Thevenin impedance is restored to its original value before DG integration. Consequently, the fault current almost returns to the base value as shown in Fig 18.

As shown in Table 18 Based on the obtained results, the following discussion summarizes the impact of integrating different distributed generation technologies on the fault-current level and the corresponding DFCL impedance required to restore the original protection coordination, the base-case configuration represents the reference operating condition in which the overcurrent relay settings are already properly coordinated; therefore, no DFCL is required. Under this condition, the total

**Table 18:** Comparison of fault-current contributions of different distributed generation technologies.

Scenario	Integrated New DG Type	Integrated DG Rating (MVA)	Total Fault current contribution from added DGs (KA)	current contribution from only new added DG (KA)	Total Fault current at PCC (KA)	Percentage of fault current contribution from new DG added	DFCL Impedance (Ohm)
**Original Network (DG1)**	Synchronous (DG1)	1.5	0.198	0.198	2.076	9.54%	No need (relays are coordinated)
**A (DG1& DG 2)**	Synchronous (DG2)	1.5	0.375	0.177	2.249	7.87%	30.01
**B (DG1& DG 2&DG3)**	Synchronous (DG3)	5	0.784	0.409	2.657	15.39%	47.47
**C** **(DG1& DG 2&DG3&WT1)**	Induction (WT1)	1.765	0.929	0.145	2.805	5.17%	50.01
**D (DG1& DG 2&DG3&WT1&WT2)**	Induction (WT2) (different internal impedance)	1.765	1.053	0.124	2.932	4.23%	51.66
**E (DG1& DG 2&DG3&WT1&WT2&PV)**	PV (with Inverter)	1	1.085	0.032	2.965	1.08%	52

fault current at the PCC is 2.076 kA, while the existing synchronous generator (DG1) contributes 0.198 kA, corresponding to 9.54% of the total fault current.

In Scenario A, integrating an additional 1.5 MVA synchronous generator (DG2) increases the DG contribution to 0.375 kA, resulting in a total fault current of 2.249 kA and a required DFCL impedance of 30.01 Ω. The newly added DG contributes 7.87% of the total fault current. This increase is due to the fault-current contribution of the additional synchronous generator, which raises the overall fault level and necessitates the installation of a DFCL to maintain protection coordination.

In Scenario B, adding a larger 5 MVA synchronous generator (DG3) further increases the DG contribution to 0.784 kA, resulting in a total fault current of 2.657 kA and a required DFCL impedance of 47.47 Ω. The new DG contribution reaches 15.39%, which is the highest among all investigated DG units. This is attributed to the larger generator rating and the inherently high fault-current capability of synchronous generators.

In Scenario C, integrating the 1.765 MVA induction generator (WT1) increases the DG contribution to 0.929 kA, producing a total fault current of 2.805 kA with a required DFCL impedance of 50.01 Ω. However, the newly added induction generator contributes only 5.17% of the total fault current, which is considerably lower than the contribution of the synchronous generator in Scenario B. This indicates that the induction generator has a lower influence on the fault level than the synchronous generator.

In Scenario D, a second induction generator (WT2) with a higher internal impedance is integrated. Although the overall DG contribution increases to 1.053 kA and the total fault current reaches 2.932 kA, the contribution of the newly added DG decreases to 4.23%. This reduction is mainly attributed to the higher internal impedance of WT2, which limits its fault-current contribution compared with WT1.

Finally, in Scenario E, a 1 MVA inverter-based PV system is added. The total fault current increases slightly to 2.965 kA, while the overall DG contribution reaches 1.085 kA, requiring a DFCL impedance of 52 Ω. Nevertheless, the contribution of the PV unit itself is only 1.08%, confirming that inverter-based DGs contribute very limited fault current because of their converter current-limiting control.

**Fig 19:**
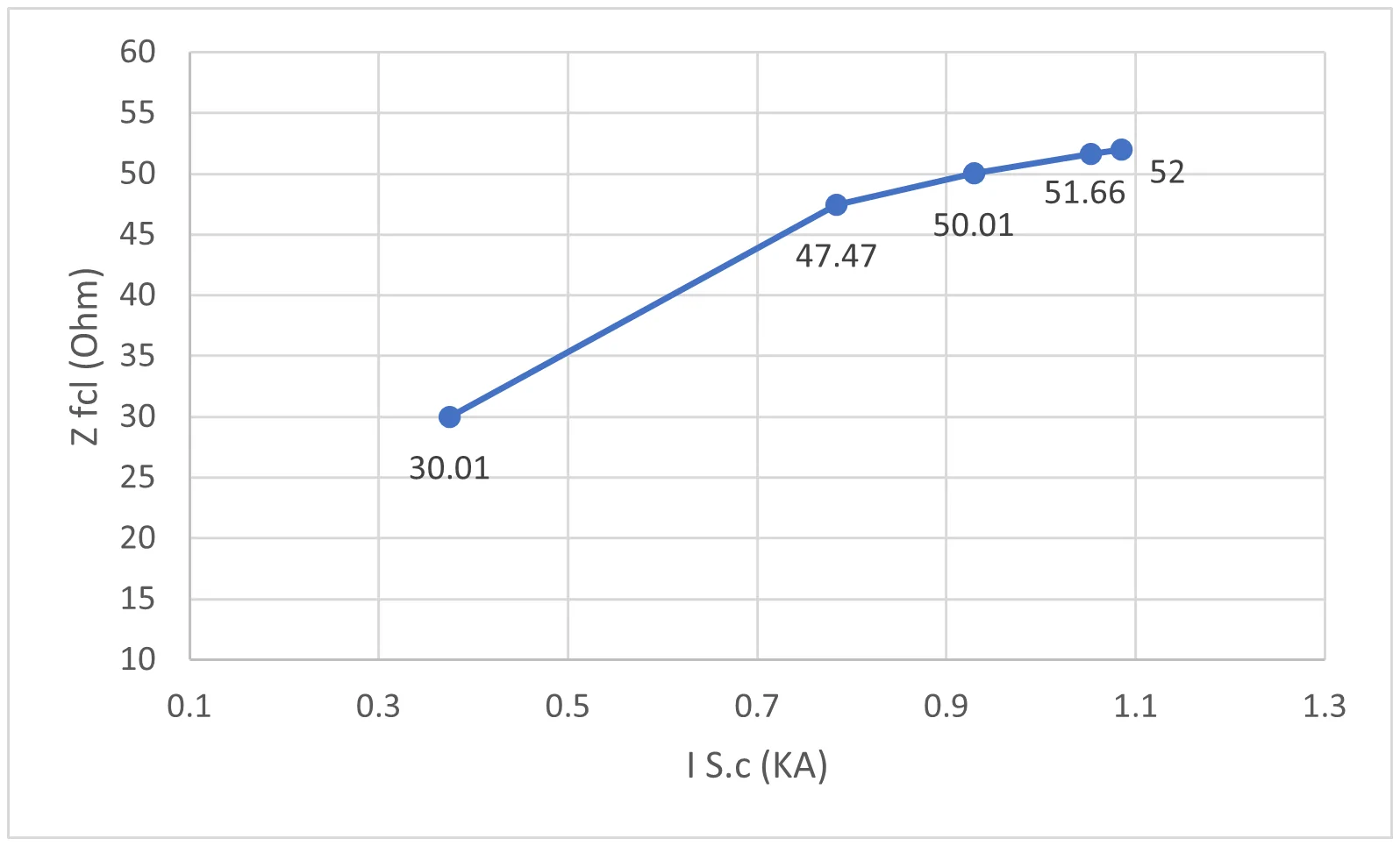
FCL Scenarios impedance vs short circuit current.

Overall, the results demonstrate that the total fault current increases progressively with each additional DG integration, leading to a corresponding increase in the required DFCL impedance as shown in Table 19 and Fig 19 from 30.01 Ω in Scenario A to 52 Ω in Scenario E. Furthermore, the fault-current contribution strongly depends on the DG technology and its electrical characteristics. Synchronous generators exhibit the highest contribution, induction generators provide lower contributions, while inverter-based PV systems have the smallest impact on the network fault level.

**Table 19:** FCL Scenarios impedance vs short circuit current.

Scenario	A	B	C	D	E
$I_{SC}$	0.375	0.784	0.929	1.053	1.085
$Z_{FCL}$	30.01	47.47	50.01	51.66	52

## 6. Conclusion

In this study, a directional fault current limiter (DFCL) is proposed to enhance protection coordination in distribution networks with distributed generations (DGs). The device is installed between the upstream and downstream sections of the network and operates selectively based on the fault location. During upstream faults, the DFCL limits the fault current contribution from the downstream network, thereby preventing excessive fault levels and preserving the coordination of upstream overcurrent relays (OCRs). In contrast, for downstream faults, the DFCL remains inactive, allowing the upstream network to fully contribute to the fault current. The coordination performance of the OCRs is evaluated based on the calculated fault current levels using the MVA Short Circuit (MVAsc) under different scenarios and operating conditions, with and without the DFCL, particularly after the integration of new DG units. The effectiveness of the proposed approach is validated through comprehensive simulation scenarios using ETAP software.

**Acknowledgement**

This article has been produced with the financial support of the European Union under the REFRESH – Research Excellence for Region Sustainability and High-tech Industries project number CZ.10.03.01/00/22_003/0000048 via the Operational Programme Just Transition.

## Supporting information

S1 File Detailed ETAP simulation reports for the investigated system configurations. This file contains the detailed ETAP short-circuit calculation reports corresponding to Figs. 8–18. The reports cover the investigated system configurations with and without the proposed fault current limiter (FCL), including configurations incorporating distributed generators (DGs), wind turbines (WTs), and photovoltaic (PV) generation. The reports provide the three-phase short-circuit results at Bus 2, including the peak short-circuit current, steady-state short-circuit current, and the individual contributions of the connected generation sources.
(XLS)

S1 Data Detailed ETAP simulation reports for the investigated system (XLS)
