# Supplementary material for: Adaptive and resilient protection coordination in smart microgrids enabled by a directional fault current limiter
Source: PLoS One. 2026 Aug 19;21(8):e0356375. doi: 10.1371/journal.pone.0356375 (PMC13489461; doi:10.1371/journal.pone.0356375)
Supplement: Supplementary file 1 [file pone.0356375.s004.docx]

**Ms. Ref. No.: PONE-D-26-25751**

**Enhanced Distance Protection for HVDC Lines Using Adaptive Neuro-Fuzzy Inference Systems**

**Dear Editors and Reviewers**

The authors sincerely thank the editor(s) and reviewers for their valuable comments and suggestions, which have greatly improved the quality and clarity of our paper. We have carefully addressed all remarks and provided detailed responses. All revisions are highlighted in red in the updated manuscript. We truly appreciate the reviewers’ time and constructive feedback.

***Journal Requirements:***

1. Please ensure that your manuscript meets PLOS ONE's style requirements, including those for file naming. The PLOS ONE style templates can be found at

https://journals.plos.org/plosone/s/file?id=wjVg/PLOSOne_formatting_sample_main_body.pdf https://journals.plos.org/plosone/s/file?id=ba62/PLOSOne_formatting_sample_title_authors_affiliations.pdf

Thank you for your kind guidance. We confirm that the manuscript has been revised and formatted in full accordance with the PLOS ONE style and file naming requirements.

2. Please note that PLOS One has specific guidelines on code sharing for submissions in which author-generated code underpins the findings in the manuscript. In these cases, we expect all author-generated code to be made available without restrictions upon publication of the work. Please review our guidelines at https://journals.plos.org/plosone/s/materials-and-software-sharing#loc-sharing-code and ensure that your code is shared in a way that follows best practice and facilitates reproducibility and reuse.

We have fully complied with the PLOS One code sharing guidelines to ensure transparency and reproducibility. Thank you for your kind guidance.

3. Thank you for stating the following in the Acknowledgments Section of your manuscript:

“This article has been produced with the financial support of the European Union under the REFRESH – Research Excellence For Region Sustainability and High-tech Industries project number CZ.10.03.01/00/22_003/0000048 via the Operational Programe Just Transition.”

We note that you have provided funding information that is not currently declared in your Funding Statement. However, funding information should not appear in the Acknowledgments section or other areas of your manuscript. We will only publish funding information present in the Funding Statement section of the online submission form.

Please remove any funding-related text from the manuscript and let us know how you would like to update your Funding Statement. Currently, your Funding Statement reads as follows:

“The author(s) received no specific funding for this work.”

Please include your amended statements within your cover letter; we will change the online submission form on your behalf.

Thank you for your careful observation. We sincerely apologize for the inconsistency between the Funding Statement in the online submission system and the funding information previously included in the manuscript.

The selection of "The author(s) received no specific funding for this work." during the previous submission was an inadvertent administrative oversight and did not reflect the actual funding status of this research.

In accordance with the journal's instructions, we have removed all funding-related text from the manuscript, including the Acknowledgments section. We have also updated the Funding Statement in the online submission system to accurately reflect the funding information.

We sincerely regret this oversight and appreciate the opportunity to correct the submission. We confirm that the discrepancy resulted solely from an unintentional administrative error during the previous submission process and did not affect the scientific content, integrity, or transparency of the manuscript.

Thank you for your understanding and consideration.

4. We note that your Data Availability Statement is currently as follows:

“All relevant data supporting the findings of this study are included within the manuscript and its supporting information files. Additional simulation data and analysis results are available from the corresponding author upon reasonable request.”

Please confirm at this time whether or not your submission contains all raw data required to replicate the results of your study. Authors must share the “minimal data set” for their submission. PLOS defines the minimal data set to consist of the data required to replicate all study findings reported in the article, as well as related metadata and methods (https://journals.plos.org/plosone/s/data-availability#loc-minimal-data-set-definition).

For example, authors should submit the following data:

- The values behind the means, standard deviations and other measures reported;

- The values used to build graphs;

- The points extracted from images for analysis.

Authors do not need to submit their entire data set if only a portion of the data was used in the reported study.

If your submission does not contain these data, please either upload them as Supporting Information files or deposit them to a stable, public repository and provide us with the relevant URLs, DOIs, or accession numbers. For a list of recommended repositories, please see https://journals.plos.org/plosone/s/recommended-repositories.

If there are ethical or legal restrictions on sharing a de-identified data set, please explain them in detail (e.g., data contain potentially sensitive information, data are owned by a third-party organization, etc.) and who has imposed them (e.g., an ethics committee). Please also provide contact information for a data access committee, ethics committee, or other institutional body to which data requests may be sent. If data are owned by a third party, please indicate how others may request data access.

Thank you for your valuable comment and for clarifying the PLOS ONE Data Availability requirements.

We appreciate this important point. To fully comply with the journal's Data Availability Policy, we will upload the complete minimal data set as Supporting Information. The uploaded files will include the numerical data underlying the figures, graphs, and analyses presented in the manuscript, together with the relevant simulation output data required to reproduce the reported results.

Accordingly, we will revise the Data Availability Statement to reflect that the minimal data set is provided as Supporting Information accompanying the manuscript

5. Please upload a new copy of Figures 2, 4, 5, 6, 8, 9, 10, and 12 as the detail is not clear. Please follow the link for more information: <https://journals.plos.org/plosone/s/figures>

We sincerely thank the reviewer for the valuable comment. The figures (5a, 5b, 7a, 7b, 12, 13, and 14a) have been improved and replaced with higher-resolution versions to ensure clarity. We highly appreciate your suggestion, which has enhanced the quality of the manuscript.

6. Please ensure that you refer to Figure 3 in your text as, if accepted, production will need this reference to link the reader to the figure

Thank you for this observation. We have revised the manuscript to include an explicit reference to **Figure 3** in the main text at the appropriate location, ensuring consistency with the journal's formatting requirements.

7. If the reviewer comments include a recommendation to cite specific previously published works, please review and evaluate these publications to determine whether they are relevant and should be cited. There is no requirement to cite these works unless the editor has indicated otherwise.

Thank the reviewer for the valuable recommendation. We carefully reviewed all the suggested references and found them relevant to the scope of the present study. Accordingly, these references have been incorporated into the revised manuscript to strengthen the literature review and better position the proposed work within the context of recent advances in microgrid protection and power electronic systems. Specifically:

[1] Dehghanpour et al. (2018), "Optimal coordination of directional overcurrent relays in microgrids by using cuckoo-linear optimization algorithm and fault current limiter," has been cited in the Introduction (Page 4, Lines 82) to support the discussion on directional overcurrent relay coordination and the application of fault current limiters in microgrids.

[2] Meng et al. (2026), "Dissipativity-Based Multiport Stability Root-Cause Identification and Mitigation for Solid-State Transformers," has been cited in the Importance of the fault current limiter (FCL) section (Page 5, Lines 111) to enrich the discussion on power electronic converter-based systems and stability considerations in modern power networks.

[3] Feng et al. (2026), "Aggregation Model and Market Mechanism for Virtual Power Plant Participation in Inertia and Primary Frequency Response," has been cited Importance of the fault current limiter (FCL) section (Page 5, Lines 111) to provide additional background on emerging grid-support technologies and the evolving role of inverter-based resources in modern power system

**Reviewer 1**

**Reviewer’s Comment:**

1.The title and abstract claim a "novel optimization methodology" that simultaneously minimizes DFCL installation cost and reliability-related cost. However, no optimization algorithm, no cost model, and no objective function appear anywhere in the main text or in any table or figure. The paper presents a Thevenin-based analytical method for impedance sizing, which is straightforward engineering calculation rather than optimization. The authors should either (a) remove the optimization language and retitle the contribution accurately, or (b) add the full cost model, objective function, and solver details that are referenced in the abstract.

**Author’s Response:**

We sincerely thank the reviewer for this valuable comment. We agree that the original wording in the abstract could be interpreted as suggesting the implementation of a formal optimization framework involving an objective function, cost model, and optimization algorithm. This was not the intention of the present work.

The proposed method is an analytical methodology for determining the appropriate DFCL impedance based on the network operating conditions, fault level restoration, and protection coordination requirements, rather than a mathematical optimization approach. Therefore, all optimization-related terminology has been removed from the abstract and throughout the manuscript to accurately reflect the actual contribution of this study. The manuscript title remains unchanged because it describes the adaptive and resilient protection coordination capability enabled by the proposed DFCL methodology, without referring to an optimization framework .

**Reviewer’s Comment:**

2. Statistical Analysis – No2. The DFCL impedance sizing formula is derived as Z_DFCL = Z_th(base) - Z_th(after DG). For Scenario A the numbers given are 64.51 ohm (base) - 34.22 ohm (after DG2) = 30.29 ohm, which matches Table 11. However, for Scenario B the Thevenin impedance after adding DG3 is listed as 16.57 ohm, yielding Z_DFCL = 47.94 ohm (Table 11), yet Table 12 reports I_SC = 0.784 kA for Scenario B, while Scenario C reports I_SC = 0.929 kA with Z_DFCL = 53.47 ohm, and Scenario D reports I_SC = 1.053 kA with Z_DFCL = 54.65 ohm -- identical to Scenario E. The identical I_SC and Z_DFCL values for Scenarios D and E (Table 12), despite D using an induction WTG and E using a PV inverter whose fault contribution the authors themselves describe as negligible, needs explicit explanation. The paper must show the per-scenario Thevenin calculation steps so readers can verify the numbers independently.

**Author’s Response:**

We sincerely thank the reviewer for this insightful comment. We agree that the previous version did not sufficiently explain the differences between the induction generator and inverter-based PV scenarios, and that the identical values reported for Scenarios D and E could lead to confusion.

To address this concern, the Thevenin equivalent impedance calculations have been presented in detail for each scenario. The input parameters, intermediate short-circuit capacity calculations, equivalent branch MVASCMVA_{SC}MVASC, and the resulting Thevenin impedance and required DFCL impedance are provided step by step.

**5.1.ORIGINAL Network configuration (1 Synchronous Generator (G1)):**

**Proposed Analytical Procedure**

The original network configuration at the downstream MG ,consists of a single synchronous generator (G1), the step-up transformer(T2) , the DG feeder impedance(ZDG1) , and the microgrid equivalent impedance (ZMG) as shown in Fig 1To determine the equivalent Thevenin impedance seen from the fault location at (Bus 2 ) which represent the point of common coupling (PCC) to study the effect of DGs contributions, all system data needed are given ,the short-circuit capacity (MVAsc) of each component is first calculated individually. These values are then combined according to their series and parallel connections to obtain the overall equivalent impedance for this configuration

For Generator (DG1)

The short-circuit capacity of the synchronous generator is calculated from its rated apparent power $\left( \boldsymbol{S} \right)$and sub transient reactance$\left( \boldsymbol{Xd}” \right)$

**MVA sc =**$\frac{\boldsymbol{S}}{\boldsymbol{Xd"}}$
$\boldsymbol{=}\frac{\boldsymbol{1.5}}{\boldsymbol{0.18}}\boldsymbol{=8.33}$ **MVA**

For Transformer (T2)

The transformer short-circuit capacity is obtained using its rated power $\left( \boldsymbol{S} \right)$and percentage impedance **(**$\boldsymbol{Z\%})$

**MVA sc =**$\frac{\boldsymbol{S}}{\boldsymbol{Z\%}}$

$$\boldsymbol{=}\frac{\boldsymbol{2}}{\boldsymbol{0.06}}\boldsymbol{=33.33 MVA}$$

For DG1 feeder & ZMG

The impedance of the DG1 feeder is converted into its equivalent short-circuit capacity using the base voltage **(**$\boldsymbol{KV})$

**MVA sc =**$\frac{\boldsymbol{KV}^{\boldsymbol{2}}}{\boldsymbol{ZDG}\boldsymbol{1}}$

$$\boldsymbol{=}\frac{\boldsymbol{20}^{\boldsymbol{2}}}{\sqrt{\boldsymbol{(0.081)}^{\boldsymbol{2}}\boldsymbol{+}\boldsymbol{(0.057)}^{\boldsymbol{2}}}}\boldsymbol{=4038.55 MVA}$$

**MVA sc =**$\frac{\boldsymbol{KV}^{\boldsymbol{2}}}{\boldsymbol{ZMG}}$

$$\boldsymbol{=}\frac{\boldsymbol{20}^{\boldsymbol{2}}}{\sqrt{\boldsymbol{(2.15)}^{\boldsymbol{2}}\boldsymbol{+}\boldsymbol{(3.24)}^{\boldsymbol{2}}}}\boldsymbol{=102.868 MVA}$$

The load branch is neglected in the short-circuit analysis because it does not contribute to the fault current, so Since the generator, transformer, and DG feeder are connected in series from the fault point perspective, their short-circuit capacities are combined using the reciprocal relationship.

**MVA (Series) =**$\frac{\boldsymbol{1}}{\frac{\boldsymbol{1}}{\sum\boldsymbol{MVA}}}$

$$\boldsymbol{=}\frac{\boldsymbol{1}}{\frac{\boldsymbol{1}}{\boldsymbol{MVA (ALL GENERATION)}}}\boldsymbol{=}\frac{\boldsymbol{1}}{\frac{\boldsymbol{1}}{\boldsymbol{8.33}}\boldsymbol{+}\frac{\boldsymbol{1}}{\boldsymbol{33.33}}\boldsymbol{+}\frac{\boldsymbol{1}}{\boldsymbol{4038.55}}}\boldsymbol{=6.65 MVA}$$

The generation branch is connected in series with the equivalent microgrid impedance. Therefore, the equivalent short-circuit capacity is first obtained using the reciprocal combination, after which it is converted into the corresponding Thevenin impedance. and this is the value of system impedance that the limiter try to adjust to restore the fault current as its desired value to achieve coordinated values

**Z =**$\frac{\boldsymbol{KV}^{\boldsymbol{2}}}{\sum\boldsymbol{MVA sc}}$
$\boldsymbol{=}\frac{\boldsymbol{20}^{\boldsymbol{2}}}{\frac{\boldsymbol{1}}{\frac{\boldsymbol{1}}{\boldsymbol{MVA(ALL GENERATION)}}\boldsymbol{+}\frac{\boldsymbol{1}}{\boldsymbol{MVA (Imp)}}}}\boldsymbol{=}\frac{\boldsymbol{20}^{\boldsymbol{2}}}{\frac{\boldsymbol{1}}{\frac{\boldsymbol{1}}{\boldsymbol{6.65}}\boldsymbol{+}\frac{\boldsymbol{1}}{\boldsymbol{102.868}}}}\boldsymbol{=64}$ **ohm**

**Scenario A:**

**Proposed Analytical Procedure**

In this scenario, integrating an additional **1.5 MVA synchronous generator (DG2),** so the microgrid contains DG1+DG2

For Generator (DG2)

The short-circuit capacity of the synchronous generator is calculated from its rated apparent power $\left( \boldsymbol{S} \right)$and sub transient reactance$\left( \boldsymbol{Xd}” \right)$

**MVA sc =**$\frac{\boldsymbol{S}}{\boldsymbol{Xd"}}$
$\boldsymbol{=}\frac{\boldsymbol{1.5}}{\boldsymbol{0.18}}\boldsymbol{=8.33}$ **MVA**

For Transformer (T3)

The transformer short-circuit capacity is obtained using its rated power $\left( \boldsymbol{S} \right)$and percentage impedance **(**$\boldsymbol{Z\%})$

**MVA sc =**$\frac{\boldsymbol{S}}{\boldsymbol{Z\%}}$

$$\boldsymbol{=}\frac{\boldsymbol{2}}{\boldsymbol{0.06}}\boldsymbol{=33.33 MVA}$$

For DG2 feeder & ZMG

The impedance of the DG2 feeder is converted into its equivalent short-circuit capacity using the base voltage **(**$\boldsymbol{KV})$

**MVA sc =**$\frac{\boldsymbol{KV}^{\boldsymbol{2}}}{\boldsymbol{Z}}$

$$\boldsymbol{=}\frac{\boldsymbol{20}^{\boldsymbol{2}}}{\sqrt{\boldsymbol{(0.162)}^{\boldsymbol{2}}\boldsymbol{+}\boldsymbol{(0.114)}^{\boldsymbol{2}}}}\boldsymbol{=2019.28 MVA}$$

**MVA sc =**$\frac{\boldsymbol{KV}^{\boldsymbol{2}}}{\boldsymbol{ZMG}}$

$$\boldsymbol{=}\frac{\boldsymbol{20}^{\boldsymbol{2}}}{\sqrt{\boldsymbol{(2.15)}^{\boldsymbol{2}}\boldsymbol{+}\boldsymbol{(3.24)}^{\boldsymbol{2}}}}\boldsymbol{=102.868 MVA}$$

The load branch is neglected in the short-circuit analysis because it does not contribute to the fault current, so Since the generator, transformer, and DG feeder are connected in series from the fault point perspective, their short-circuit capacities are combined using the reciprocal relationship.

**MVA(Series) =**$\frac{\boldsymbol{1}}{\frac{\boldsymbol{1}}{\sum\boldsymbol{MVA}}}$

$$\boldsymbol{=}\frac{\boldsymbol{1}}{\frac{\boldsymbol{1}}{\boldsymbol{MVA (ALL GENERATION)}}}\boldsymbol{=}\frac{\boldsymbol{1}}{\frac{\boldsymbol{1}}{\boldsymbol{8.33}}\boldsymbol{+}\frac{\boldsymbol{1}}{\boldsymbol{33.33}}\boldsymbol{+}\frac{\boldsymbol{1}}{\boldsymbol{2019.28}}}\boldsymbol{=6.64 MVA}$$

The generation branch is connected in series with the equivalent microgrid impedance. Therefore, the equivalent short-circuit capacity is first obtained using the reciprocal combination, after which it is converted into the corresponding Thevenin impedance. and this is the value of system impedance that the limiter try to adjust to restore the fault current as its desired value to achieve coordinated values

**Z =**$\frac{\boldsymbol{KV}^{\boldsymbol{2}}}{\sum\boldsymbol{MVA sc}}$

$$\boldsymbol{=}\frac{\boldsymbol{20}^{\boldsymbol{2}}}{\frac{\boldsymbol{1}}{\frac{\boldsymbol{1}}{\boldsymbol{MVA(ALL GENERATION)}}\boldsymbol{+}\frac{\boldsymbol{1}}{\boldsymbol{MVA (Imp)}}}}\boldsymbol{=}\frac{\boldsymbol{20}^{\boldsymbol{2}}}{\frac{\boldsymbol{1}}{\frac{\boldsymbol{1}}{\boldsymbol{6.64+6.65}}\boldsymbol{+}\frac{\boldsymbol{1}}{\boldsymbol{102.868}}}}\boldsymbol{=33.99}$$

The FCL impedance is determined by the difference between the scenario impedance and the original system impedance, thereby restoring the system to its original impedance level.

FCL impedance = Scenario impedance − Original impedance = 64 – 33.99 = **30.01 Ohm**

**DFCL Impedance Validation:**

Before adding DG2 , the contribution of the microgrid as shown in Fig 1 with only DG1 was about 0.198 kA, the coordination time values and parameters were adjusted according to this case for the entire up and down network ,due to adding a new generation source this will affect the fault current by increasing its value because the contribution from the microgrid will be increased.

Fig 1. MG contribution with DG1 (original state)

As shown in Table 2 and Fig 2, integrating an additional **1.5 MVA synchronous generator (DG2)** increases the DG contribution to **0.375 kA**, resulting in a total fault current of **2.249 kA**, this leads to miscoordination at BUS-3 and BUS-4 because those buses are located at the upstream network which the contribution current will flow through.

Table 1. Original relay coordination with the microgrid

| **Fault Location** | **Relay unit (primary, back-up)** | **Relay current (kA)** | **Operating time (s)** | **CTI (s)** |
| --- | --- | --- | --- | --- |
| **BUS 3** | OCR 2 (Primary) | 1.181 | 0.321 | 0.208 |
|  | OCR 1 (Back-up) | 1.075 | 0.529 |  |
| **BUS 4** | OCR 3 (Primary) | 0.794 | 0.532 | 0.219 |
|  | OCR 2 (Back-up) | 0.794 | 0.751 |  |
| **BUS 6** | OCR 5 (Primary) | 0.474 | 0.189 | 0.327 |
|  | OCR 4 (Back-up) | 0.409 | 0.516 |  |
| **BUS DG1** | OCR 6 (Primary) | 1.19 | 0.106 | 0.278 |
|  | OCR 4 (Back-up) | 1.19 | 0.384 |  |

Table 2. FAULT CURRENT RESULTS FOR THE NETWORK CONNECTED TO DG1&DG2

| **Fault Location** | **Relay unit (primary, back-up)** | **Relay current (kA)** | **Operating time (s)** | **CTI (s)** |
| --- | --- | --- | --- | --- |
| BUS 3 | OCR 2 (Primary) | 1.233 | 0.302 | 0.898 |
|  | OCR 1 (Back-up) | 1.041 | 1.2 |  |
| BUS 4 | OCR 3 (Primary) | 0.818 | 0.509 | 0.175 |
|  | OCR 2 (Back-up) | 0.818 | 0.684 |  |
| BUS 6 | OCR 5 (Primary) | 0.493 | 0.189 | 0.328 |
|  | OCR 4 (Back-up) | 0.376 | 0.547 |  |
| BUS DG1 | OCR 6 (Primary) | 1.367 | 0.105 | 0.279 |
|  | OCR 4 (Back-up) | 1.188 | 0.384 |  |

Fig 2. MG contribution after adding DG2

The newly added DG contributes **7.87%** of the total fault current. Accordingly, a DFCL with a calculated impedance of **30.01 Ω** is required only for upstream faults, where it limits the microgrid fault-current contribution to the utility grid. For downstream faults as shown in Fig 3 , the DFCL is intentionally blocked since the microgrid protection coordination has already been established without current limitation, and the upstream utility contribution remains constant. Operating the DFCL under downstream fault conditions would unnecessarily reduce the fault current, potentially leading to protection miscoordination. Hence, directional control is indispensable to ensure that the DFCL is activated exclusively during upstream faults, as verified in Tables 3 and 4.

Table 3. Relay coordination after adding None-DFCL

| **Fault Location** | **Relay unit (primary, back-up)** | **Relay current (kA)** | **Operating time (s)** | **CTI (s)** |
| --- | --- | --- | --- | --- |
| **BUS 3** | OCR 2 (Primary) | 1.181 | 0.321 | 0.208 |
|  | OCR 1 (Back-up) | 1.076 | 0.529 |  |
| **BUS 4** | OCR 3 (Primary) | 0.794 | 0.533 | 0.218 |
|  | OCR 2 (Back-up) | 0.794 | 0.751 |  |
| **BUS 6** | OCR 5 (Primary) | 0.354 | 0.189 | 1.382 |
|  | OCR 4 (Back-up) | 0.157 | 1.571 |  |
| **BUS DG1** | OCR 6 (Primary) | 0.512 | 0.123 | 0.504 |
|  | OCR 4 (Back-up) | 0.318 | 0.627 |  |

Table 4. Relay coordination after adding DFCL

| **Fault Location** | **Relay unit (Primary,Back-up)** | **Relay current (kA)** | **Operating time (s)** | **CTI (s)** |
| --- | --- | --- | --- | --- |
| **BUS 3** | OCR 2 (Primary) | 1.181 | 0.321 | 0.208 |
|  | OCR 1 (Back-up) | 1.076 | 0.529 |  |
| **BUS 4** | OCR 3 (Primary) | 0.794 | 0.533 | 0.218 |
|  | OCR 2 (Back-up) | 0.794 | 0.751 |  |
| **BUS 6** | OCR 5 (Primary) | 0.493 | 0.189 | 0.328 |
|  | OCR 4 (Back-up) | 0.376 | 0.517 |  |
| **BUS DG1** | OCR 6 (Primary) | 1.367 | 0.105 | 0.279 |
|  | OCR 4 (Back-up) | 1.188 | 0.384 |  |

Fig 3. Restoration of the system parameters by Implement DFCL at PCC

**5.2. Scenario B:
Proposed Analytical Procedure**

In this scenario, adding a larger **5 MVA synchronous generator (DG3)** ,so the microgrid contains (DG1+DG2+DG3)

For Generator (DG3)

The short-circuit capacity of the synchronous generator is calculated from its rated apparent power $\left( \boldsymbol{S} \right)$and sub transient reactance$\left( \boldsymbol{Xd}” \right)$

**MVA sc (G3) =**$\frac{\boldsymbol{S}}{\boldsymbol{Xd"}}$
$\boldsymbol{=}\frac{\boldsymbol{5}}{\boldsymbol{0.18}}\boldsymbol{=27.78}$ **MVA**

For Transformer (T5)

The transformer short-circuit capacity is obtained using its rated power $\left( \boldsymbol{S} \right)$and percentage impedance **(**$\boldsymbol{Z\%})$

**MVA sc (TR2,3) =**$\frac{\boldsymbol{S}}{\boldsymbol{Z\%}}$

$$\boldsymbol{=}\frac{\boldsymbol{4}}{\boldsymbol{0.0715}}\boldsymbol{=55.94 MVA}$$

For DG3 feeder & ZMG

The impedance of the DG3 feeder is converted into its equivalent short-circuit capacity using the base voltage **(**$\boldsymbol{KV})$

**MVA sc =**$\frac{\boldsymbol{KV}^{\boldsymbol{2}}}{\boldsymbol{Z}}$

$$\boldsymbol{=}\frac{\boldsymbol{20}^{\boldsymbol{2}}}{\sqrt{\boldsymbol{(0.2)}^{\boldsymbol{2}}\boldsymbol{+}\boldsymbol{(0.15)}^{\boldsymbol{2}}}}\boldsymbol{=1600 MVA}$$

**MVA sc =**$\frac{\boldsymbol{KV}^{\boldsymbol{2}}}{\boldsymbol{ZMG}}$

$$\boldsymbol{=}\frac{\boldsymbol{20}^{\boldsymbol{2}}}{\sqrt{\boldsymbol{(2.15)}^{\boldsymbol{2}}\boldsymbol{+}\boldsymbol{(3.24)}^{\boldsymbol{2}}}}\boldsymbol{=102.868 MVA}$$

The load branch is neglected in the short-circuit analysis because it does not contribute to the fault current, so Since the generator, transformer, and DG feeder are connected in series from the fault point perspective, their short-circuit capacities are combined using the reciprocal relationship.

**MVA(Series) =**$\frac{\boldsymbol{1}}{\frac{\boldsymbol{1}}{\sum\boldsymbol{MVA}}}$

$$\boldsymbol{=}\frac{\boldsymbol{1}}{\frac{\boldsymbol{1}}{\boldsymbol{MVA (ALL GENERATION)}}}\boldsymbol{=}\frac{\boldsymbol{1}}{\frac{\boldsymbol{1}}{\boldsymbol{27.78}}\boldsymbol{+}\frac{\boldsymbol{1}}{\boldsymbol{55.94}}\boldsymbol{+}\frac{\boldsymbol{1}}{\boldsymbol{1600}}}\boldsymbol{=18.35 MVA}$$

The generation branch is connected in series with the equivalent microgrid impedance. Therefore, the equivalent short-circuit capacity is first obtained using the reciprocal combination, after which it is converted into the corresponding Thevenin impedance. and this is the value of system impedance that the limiter try to adjust to restore the fault current as its desired value to achieve coordinated values

**Z =**$\frac{\boldsymbol{KV}^{\boldsymbol{2}}}{\sum\boldsymbol{MVA sc}}$

$$\boldsymbol{=}\frac{\boldsymbol{20}^{\boldsymbol{2}}}{\frac{\boldsymbol{1}}{\frac{\boldsymbol{1}}{\boldsymbol{MVA(ALL GENERATION)}}\boldsymbol{+}\frac{\boldsymbol{1}}{\boldsymbol{MVA (Imp)}}}}\boldsymbol{=}\frac{\boldsymbol{20}^{\boldsymbol{2}}}{\frac{\boldsymbol{1}}{\frac{\boldsymbol{1}}{\boldsymbol{6.65+6.64+18.35}}\boldsymbol{+}\frac{\boldsymbol{1}}{\boldsymbol{102.868}}}}\boldsymbol{=16.53}$$

The FCL impedance is determined by the difference between the scenario impedance and the original system impedance, thereby restoring the system to its original impedance level.

FCL impedance = Scenario impedance − Original impedance = 64-16.53= **47.47 Ohm**

**DFCL Impedance Validation:**

Adding a larger **5 MVA synchronous generator (DG3)** , as shown in Fig 4 and Table 5, the contribution current turned from 0.375 KA to 0.784 KA resulting in a total fault current of **2.657 kA**

As shown in table 8 Bus-4 and Bus-3 which are the upstream buses will be affected comparing to the original values shown in Table 1 , but the coordination of the downstream microgrid will be accepted, by Following the detailed analysis presented for Scenario A, the same MVAsc-based methodology is applied to this one to get the suitable impedance for the DFCL shown in Fig 5, we will be able to restore the fault current as the base value

Fig 4. MG contribution after adding DG3

Table 5. Relay coordination after adding DG 3

| **Fault Location** | **Relay unit (primary, back-up)** | **Relay current (kA)** | **Operating time (s)** | **CTI (s)** |
| --- | --- | --- | --- | --- |
| **BUS 3** | OCR 2 (Primary) | 1.352 | 0.269 | 0.256 |
|  | OCR 1 (Back-up) | 0.96 | 0.525 |  |
| **BUS 4** | OCR 3 (Primary) | 0.868 | 0.463 | 0.113 |
|  | OCR 2 (Back-up) | 0.868 | 0.576 |  |
| **BUS 6** | OCR 5 (Primary) | 0.539 | 0.189 | 0.474 |
|  | OCR 4 (Back-up) | 0.3 | 0.663 |  |
| **BUS DG1** | OCR 6 (Primary) | 1.932 | 0.105 | 0.279 |
|  | OCR 4 (Back-up) | 1.185 | 0.384 |  |

Fig 5. Restoration of the system parameters by Implement DFCL at PCC

**5.3 Scenario C:**

**Proposed Analytical Procedure**

In this scenario, integrating the 1.765 MVA induction generator (WT1), so the microgrid contains (DG1+DG2+DG3+WT1)

For Induction Generator (WT1)

The short-circuit capacity of the induction wind turbine generator is calculated based on its rated apparent power $\left( \boldsymbol{S} \right)$and equivalent reactance $\left( X_{eq} \right)$

**MVA sc (WT1) =**$\frac{\boldsymbol{S}}{\boldsymbol{Xeq}}$
$\boldsymbol{=}\frac{\boldsymbol{1.765}}{\boldsymbol{0.16667}}\boldsymbol{=10.59}$ **MVA**

For Transformer (T6)

The transformer short-circuit capacity is obtained using its rated power $\left( \boldsymbol{S} \right)$and percentage impedance **(**$\boldsymbol{Z\%})$

**MVA sc (TR6) =**$\frac{\boldsymbol{S}}{\boldsymbol{Z\%}}$

$$\boldsymbol{=}\frac{\boldsymbol{2}}{\boldsymbol{0.06}}\boldsymbol{=33.33 MVA}$$

For WT1 feeder & ZMG

The impedance of the WT1 feeder is converted into its equivalent short-circuit capacity using the base voltage **(**$\boldsymbol{KV})$

**MVA sc =**$\frac{\boldsymbol{KV}^{\boldsymbol{2}}}{\boldsymbol{Z}}$

$$\boldsymbol{=}\frac{\boldsymbol{20}^{\boldsymbol{2}}}{\sqrt{\boldsymbol{(0.4)}^{\boldsymbol{2}}\boldsymbol{+}\boldsymbol{(0.315)}^{\boldsymbol{2}}}}\boldsymbol{=785.64 MVA}$$

**MVA sc =**$\frac{\boldsymbol{KV}^{\boldsymbol{2}}}{\boldsymbol{ZMG}}$

$$\boldsymbol{=}\frac{\boldsymbol{20}^{\boldsymbol{2}}}{\sqrt{\boldsymbol{(2.15)}^{\boldsymbol{2}}\boldsymbol{+}\boldsymbol{(3.24)}^{\boldsymbol{2}}}}\boldsymbol{=102.868 MVA}$$

The load branch is neglected in the short-circuit analysis because it does not contribute to the fault current, so Since the WT, transformer, and WT feeder are connected in series from the fault point perspective, their short-circuit capacities are combined using the reciprocal relationship.

**MVA(Series) =**$\frac{\boldsymbol{1}}{\frac{\boldsymbol{1}}{\sum\boldsymbol{MVA}}}$

$$\boldsymbol{=}\frac{\boldsymbol{1}}{\frac{\boldsymbol{1}}{\boldsymbol{MVA (ALL GENERATION)}}}\boldsymbol{=}\frac{\boldsymbol{1}}{\frac{\boldsymbol{1}}{\boldsymbol{10.59}}\boldsymbol{+}\frac{\boldsymbol{1}}{\boldsymbol{33.33}}\boldsymbol{+}\frac{\boldsymbol{1}}{\boldsymbol{785.64}}}\boldsymbol{=7.96 MVA}$$

The generation branch is connected in series with the equivalent microgrid impedance. Therefore, the equivalent short-circuit capacity is first obtained using the reciprocal combination, after which it is converted into the corresponding Thevenin impedance. and this is the value of system impedance that the limiter try to adjust to restore the fault current as its desired value to achieve coordinated values

**Z =**$\frac{\boldsymbol{KV}^{\boldsymbol{2}}}{\sum\boldsymbol{MVA sc}}$

$$\boldsymbol{=}\frac{\boldsymbol{20}^{\boldsymbol{2}}}{\frac{\boldsymbol{1}}{\frac{\boldsymbol{1}}{\boldsymbol{MVA(ALL GENERATION)}}\boldsymbol{+}\frac{\boldsymbol{1}}{\boldsymbol{MVA (Imp)}}}}\boldsymbol{=}\frac{\boldsymbol{20}^{\boldsymbol{2}}}{\frac{\boldsymbol{1}}{\frac{\boldsymbol{1}}{\boldsymbol{6.65+6.64+18.35+7.96}}\boldsymbol{+}\frac{\boldsymbol{1}}{\boldsymbol{102.868}}}}\boldsymbol{=13.99}$$

The FCL impedance is determined by the difference between the scenario impedance and the original system impedance, thereby restoring the system to its original impedance level.

FCL impedance = Scenario impedance − Original impedance = 64-13.99= 50.01 Ohm

**DFCL Impedance Validation:**

integrating a 1.765 MVA induction generator (WT1) connected to bus-5 through step up transformer, this will lead to increase the contribution of the microgrid so this will lead to a miscoordination and increase the DG contribution to 0.929 kA, as shown in Fig.6 producing a total fault current of 2.805 kA

Fig 6. MG contribution after adding WT1

Table 6. Relay coordination after adding WT1

| **Fault Location** | **Relay unit (primary, back-up)** | **Relay current (kA)** | **Operating time (s)** | **CTI (s)** |
| --- | --- | --- | --- | --- |
| **BUS 3** | OCR 2 (Primary) | 1.388 | 0.261 | 1.156 |
|  | OCR 1 (Back-up) | 0.935 | 1.42 |  |
| **BUS 4** | OCR 3 (Primary) | 0.883 | 0.451 | 0.1 |
|  | OCR 2 (Back-up) | 0.883 | 0.552 |  |
| **BUS 6** | OCR 5 (Primary) | 0.554 | 0.189 | 0.461 |
|  | OCR 4 (Back-up) | 0.276 | 0.65 |  |
| **BUS DG1** | OCR 6 (Primary) | 2.178 | 0.105 | 0.279 |
|  | OCR 4 (Back-up) | 1.183 | 0.384 |  |

As shown in Table 6 ,Bus-4 and Bus-3 which are the upstream buses will be affected comparing to the original values at Table 1, but the coordination of the downstream microgrid will be accepted, by with a Calculated DFCL impedance of 50.01 Ω shown in Fig 7, we will be able to restore the fault currents almost to the base value.

Fig 7. Restoration of the system parameters by Implement DFCL at PCC

**5.4.Scenario D:**

**Proposed Analytical Procedure**

In this scenario, a second induction generator (**WT2**) with a **higher internal impedance** is integrated, so the microgrid contains (DG1+DG2+DG3+WT1+WT2)

For Generator (WT2)

The short-circuit capacity of the induction wind turbine generator is calculated based on its rated apparent power $\left( \boldsymbol{S} \right)$and equivalent reactance $\left( X_{eq} \right)$

**MVA sc (WT1) =**$\frac{\boldsymbol{S}}{\boldsymbol{Xeq}}$
$\boldsymbol{=}\frac{\boldsymbol{1.765}}{\boldsymbol{0.1733}}\boldsymbol{=10.185}$ **MVA**

For Transformer (T7)

The transformer short-circuit capacity is obtained using its rated power $\left( \boldsymbol{S} \right)$and percentage impedance **(**$\boldsymbol{Z\%})$

**MVA sc (TR6) =**$\frac{\boldsymbol{S}}{\boldsymbol{Z\%}}$

$$\boldsymbol{=}\frac{\boldsymbol{2}}{\boldsymbol{0.06}}\boldsymbol{=33.33 MVA}$$

For WT2 feeder & ZMG

The impedance of the WT2 feeder is converted into its equivalent short-circuit capacity using the base voltage **(**$\boldsymbol{KV})$

**MVA sc =**$\frac{\boldsymbol{KV}^{\boldsymbol{2}}}{\boldsymbol{Z}}$

$$\boldsymbol{=}\frac{\boldsymbol{20}^{\boldsymbol{2}}}{\sqrt{\boldsymbol{(0.4)}^{\boldsymbol{2}}\boldsymbol{+}\boldsymbol{(0.315)}^{\boldsymbol{2}}}}\boldsymbol{=785.64 MVA}$$

**MVA sc =**$\frac{\boldsymbol{KV}^{\boldsymbol{2}}}{\boldsymbol{ZMG}}$

$$\boldsymbol{=}\frac{\boldsymbol{20}^{\boldsymbol{2}}}{\sqrt{\boldsymbol{(2.15)}^{\boldsymbol{2}}\boldsymbol{+}\boldsymbol{(3.24)}^{\boldsymbol{2}}}}\boldsymbol{=102.868 MVA}$$

The load branch is neglected in the short-circuit analysis because it does not contribute to the fault current, so Since the WT, transformer, and WT feeder are connected in series from the fault point perspective, their short-circuit capacities are combined using the reciprocal relationship.

**MVA(Series) =**$\frac{\boldsymbol{1}}{\frac{\boldsymbol{1}}{\sum\boldsymbol{MVA}}}$

$$\boldsymbol{=}\frac{\boldsymbol{1}}{\frac{\boldsymbol{1}}{\boldsymbol{MVA (ALL GENERATION)}}}\boldsymbol{=}\frac{\boldsymbol{1}}{\frac{\boldsymbol{1}}{\boldsymbol{10.52}}\boldsymbol{+}\frac{\boldsymbol{1}}{\boldsymbol{32}}\boldsymbol{+}\frac{\boldsymbol{1}}{\boldsymbol{785.64}}}\boldsymbol{=7.72 MVA}$$

The generation branch is connected in series with the equivalent microgrid impedance. Therefore, the equivalent short-circuit capacity is first obtained using the reciprocal combination, after which it is converted into the corresponding Thevenin impedance. and this is the value of system impedance that the limiter try to adjust to restore the fault current as its desired value to achieve coordinated values

**TOTAL SCENARIO IMPEDANCE**

**Z =**$\frac{\boldsymbol{KV}^{\boldsymbol{2}}}{\sum\boldsymbol{MVA sc}}$

$$\boldsymbol{=}\frac{\boldsymbol{20}^{\boldsymbol{2}}}{\frac{\boldsymbol{1}}{\frac{\boldsymbol{1}}{\boldsymbol{MVA(ALL GENERATION)}}\boldsymbol{+}\frac{\boldsymbol{1}}{\boldsymbol{MVA (Imp)}}}}\boldsymbol{=}\frac{\boldsymbol{20}^{\boldsymbol{2}}}{\frac{\boldsymbol{1}}{\frac{\boldsymbol{1}}{\boldsymbol{6.65+6.64+18.35+7.96+7.72}}\boldsymbol{+}\frac{\boldsymbol{1}}{\boldsymbol{102.868}}}}\boldsymbol{=12.34}$$

The FCL impedance is determined by the difference between the scenario impedance and the original system impedance, thereby restoring the system to its original impedance level.

FCL impedance = Scenario impedance − Original impedance = 64-12.34= **51.66 Ohm**

**DFCL Impedance Validation:**

second induction generator (**WT2**) with a **higher internal impedance** is integrated. As shown in Fig 15 the overall DG contribution increases to **1.053 kA** and the total fault current reaches **2.932 kA**,.

As shown in Table 7 ,Bus-4 and Bus-3 which are the upstream buses will be affected comparing to the original values at Table 4, but the coordination of the downstream microgrid will be accepted, by with a Calculated DFCL impedance of **51.66 Ω** shown in Fig 8, we will be able to restore the fault currents almost to the base value.

Table 7. Relay coordination after adding WT2

| **Fault Location** | **Relay unit (primary, back-up)** | **Relay current (kA)** | **Operating time (s)** | **CTI (s)** |
| --- | --- | --- | --- | --- |
| **BUS 3** | OCR 2 (Primary) | 1.388 | 0.261 | 1.156 |
|  | OCR 1 (Back-up) | 0.935 | 1.42 |  |
| **BUS 4** | OCR 3 (Primary) | 0.883 | 0.451 | 0.1 |
|  | OCR 2 (Back-up) | 0.883 | 0.552 |  |
| **BUS 6** | OCR 5 (Primary) | 0.554 | 0.189 | 0.461 |
|  | OCR 4 (Back-up) | 0.276 | 0.65 |  |
| **BUS DG1** | OCR 6 (Primary) | 2.178 | 0.105 | 0.279 |
|  | OCR 4 (Back-up) | 1.183 | 0.384 |  |

Fig 9. Restoration of the system parameters by Implement DFCL at PCC

Fig 8. MG contribution after adding WT2

**5.5. Scenario E:**

**Proposed Analytical Procedure**

1 MVA inverter-based PV system is added. The total fault current increases slightly to 2.965 kA, while the overall DG contribution reaches 1.085 kA ,the integration of solar energy systems interfaced through power electronic converters introduces a fundamentally different behavior compared to conventional rotating generators during fault conditions. Unlike synchronous or induction machines, inverter-based distributed generation (DG) units As Solar Panels are inherently controlled and typically designed with current-limiting control strategies that restrict their output current to a value close to the rated current, even under short-circuit conditions. As a result, their fault current contribution is considerably lower than that of conventional generators, which are capable of delivering only several times their rated current during the sub-transient period. Moreover, Several studies in the literature have confirmed that photovoltaic (PV) systems contribute limited fault current, typically in the range of 1.5 to 2 times of their rated current , depending on the inverter control scheme. In our study we used to get the worst case which consider to be twice the rated current to study the impact of the PV integration.

For PV inverter :

Unlike synchronous and induction generators, inverter-based photovoltaic (PV) systems have a limited fault current contribution due to the current-limiting capability of the power electronic converter as shown in fig 10 ,the total fault current increases slightly to **2.965 kA**. Without causing a miscoordination if we used the DFCL impedance in previous scenario due to its small effect will , the short-circuit capacity of a PV unit is commonly estimated based on the inverter apparent power rating and its maximum short-circuit current capability. Accordingly, the short-circuit capacity of the PV inverter can be calculated as follows:

**MVA sc(PV) =**$\boldsymbol{S}_{\boldsymbol{inverter}}$ **x**$\boldsymbol{I}_{\boldsymbol{SC (inverter)}}$

For the studied PV unit, the inverter apparent power rating is 1 MVA, while the maximum short-circuit current is assumed to be twice the rated current

**MVA sc(PV)** $\boldsymbol{=1} \boldsymbol{x}\boldsymbol{2=2 MV}$

**MVA(Series) =**$\frac{\boldsymbol{1}}{\frac{\boldsymbol{1}}{\sum\boldsymbol{MVA}}}$

$$\boldsymbol{=}\frac{\boldsymbol{1}}{\frac{\boldsymbol{1}}{\boldsymbol{MVA (ALL GENERATION)}}}\boldsymbol{=}\frac{\boldsymbol{1}}{\frac{\boldsymbol{1}}{\boldsymbol{10.52}}\boldsymbol{+}\frac{\boldsymbol{1}}{\boldsymbol{32}}\boldsymbol{+}\frac{\boldsymbol{1}}{\boldsymbol{785.64}}}\boldsymbol{=7.72 MVA}$$

**TOTAL SCENARIO IMPEDANCE**

**Z =**$\frac{\boldsymbol{KV}^{\boldsymbol{2}}}{\sum\boldsymbol{MVA sc}}$

$$\boldsymbol{=}\frac{\boldsymbol{20}^{\boldsymbol{2}}}{\frac{\boldsymbol{1}}{\frac{\boldsymbol{1}}{\boldsymbol{MVA(ALL GENERATION)}}\boldsymbol{+}\frac{\boldsymbol{1}}{\boldsymbol{MVA (Imp)}}}}\boldsymbol{=}\frac{\boldsymbol{20}^{\boldsymbol{2}}}{\frac{\boldsymbol{1}}{\frac{\boldsymbol{1}}{\boldsymbol{6.65+6.64+18.35+7.96+7.72+2}}\boldsymbol{+}\frac{\boldsymbol{1}}{\boldsymbol{102.868}}}}\boldsymbol{=12}$$

Fig 10. MG contribution after adding PV

The FCL impedance is determined by the difference between the scenario impedance and the original system impedance, thereby restoring the system almost to its original short circuit level as shown in Fig 11.

FCL impedance = Scenario impedance − Original impedance = 64-12= 52 Ohm

Table 8. Relay coordination after adding PV

| **Fault Location** | **Relay unit (primary, back-up)** | **Relay current (kA)** | **Operating time (s)** | **CTI (s)** |
| --- | --- | --- | --- | --- |
| **BUS 3** | OCR 2 (Primary) | 1.183 | 0.320 | 0.209 |
|  | OCR 1 (Back-up) | 1.074 | 0.529 |  |
| **BUS 4** | OCR 3 (Primary) | 0.795 | 0.531 | 0.217 |
|  | OCR 2 (Back-up) | 0.795 | 0.748 |  |
| **BUS 6** | OCR 5 (Primary) | 0.510 | 0.189 | 0.461 |
|  | OCR 4 (Back-up) | 0.059 | 0.65 |  |
| **BUS DG1** | OCR 6 (Primary) | 1.529 | 0.105 | 0.279 |
|  | OCR 4 (Back-up) | 0.206 | 0.384 |  |

Fig 11. Restoration of the system parameters by Implement DFCL at PCC

Based on the obtained results, the following discussion summarizes the impact of integrating different distributed generation technologies on the fault-current level and the corresponding DFCL impedance required to restore the original protection coordination as shown in Table 9.

The base-case configuration represents the reference operating condition in which the overcurrent relay settings are already properly coordinated; therefore, no DFCL is required. Under this condition, the total fault current at the PCC is 2.076 kA, while the existing synchronous generator (DG1) contributes 0.198 kA, corresponding to 9.54% of the total fault current.

In Scenario A, integrating an additional 1.5 MVA synchronous generator (DG2) increases the DG contribution to 0.375 kA, resulting in a total fault current of 2.249 kA and a required DFCL impedance of 30.01 Ω. The newly added DG contributes 7.87% of the total fault current. This increase is due to the fault-current contribution of the additional synchronous generator, which raises the overall fault level and necessitates the installation of a DFCL to maintain protection coordination.

In Scenario B, adding a larger 5 MVA synchronous generator (DG3) further increases the DG contribution to 0.784 kA, resulting in a total fault current of 2.657 kA and a required DFCL impedance of 47.47 Ω. The new DG contribution reaches 15.39%, which is the highest among all investigated DG units. This is attributed to the larger generator rating and the inherently high fault-current capability of synchronous generators.

In Scenario C, integrating the 1.765 MVA induction generator (WT1) increases the DG contribution to 0.929 kA, producing a total fault current of 2.805 kA with a required DFCL impedance of 50.01 Ω. However, the newly added induction generator contributes only 5.17% of the total fault current, which is considerably lower than the contribution of the synchronous generator in Scenario B. This indicates that the induction generator has a lower influence on the fault level than the synchronous generator.

In Scenario D, a second induction generator (WT2) with a higher internal impedance is integrated. Although the overall DG contribution increases to 1.053 kA and the total fault current reaches 2.932 kA, the contribution of the newly added DG decreases to 4.23%. This reduction is mainly attributed to the higher internal impedance of WT2, which limits its fault-current contribution compared with WT1.

Finally, in Scenario E, a 1 MVA inverter-based PV system is added. The total fault current increases slightly to 2.965 kA, while the overall DG contribution reaches 1.085 kA, requiring a DFCL impedance of 52 Ω. Nevertheless, the contribution of the PV unit itself is only 1.08%, confirming that inverter-based DGs contribute very limited fault current because of their converter current-limiting control.

Overall, the results demonstrate that the total fault current increases progressively with each additional DG integration, leading to a corresponding increase in the required DFCL impedance as shown in Table 10 and Fig 12 from 30.01 Ω in Scenario A to 52 Ω in Scenario E. Furthermore, the fault-current contribution strongly depends on the DG technology and its electrical characteristics. Synchronous generators exhibit the highest contribution, induction generators provide lower contributions, while inverter-based PV systems have the smallest impact on the network fault level.

Table 9. Comparison of Fault-Current Contributions of Different Distributed Generation Technologies

| **Scenario** | **Integrated New DG Type** | **Integrated DG Rating (MVA)** | **Total Fault current contribution from added DGs (KA)** | **current contribution from only new added DG (KA)** | **Total Fault current at PCC (KA)** | **Percentage of fault current contribution from new DG added** | **DFCL Impedance (Ohm)** |
| --- | --- | --- | --- | --- | --- | --- | --- |
| **Original Network (DG1)** | Synchronous (DG1) | 1.5 | 0.198 | 0.198 | 2.076 | 9.54 % | No need (relays are coordinated) |
| **A  (DG1& DG 2)** | Synchronous (DG2) | 1.5 | 0.375 | 0.177 | 2.249 | 7.87 % | 30.01 |
| **B**  **(DG1& DG2&DG3)** | Synchronous (DG3) | 5 | 0.784 | 0.409 | 2.657 | 15.39 % | 47.47 |
| **C**  **(DG1& DG 2&DG3&WT1)** | Induction (WT1) | 1.765 | 0.929 | 0.145 | 2.805 | 5.17 % | 50.01 |
| **D  (DG1& DG 2&DG3&WT1&WT2)** | Induction (WT2) (different internal impedance) | 1.765 | 1.053 | 0.124 | 2.932 | 4.23 % | 51.66 |
| **E  (DG1& DG 2&DG3&WT1&WT2&PV)** | PV (with Inverter) | 1 | 1.085 | 0.032 | 2.965 | 1.08 % | 52 |

Table 10. FCL Scenarios impedance vs Short circuit current

| **Scenario** | **A** | **B** | **C** | **D** | **E** |
| --- | --- | --- | --- | --- | --- |
| $\boldsymbol{I}_{\boldsymbol{SC}}$ | 0.375 | 0.784 | 0.929 | 1.053 | 1.085 |
| $\boldsymbol{Z}_{\boldsymbol{FCL}}$ | 30.01 | 47.47 | 50.01 | 51.66 | 52 |

Fig 12. FCL Scenarios impedance vs Short circuit current

| I S.c (KA) | Z fcl (Ohm) |
| --- | --- |
| 0.375 | 30.01 |
| 0.784 | 47.47 |
| 0.929 | 50.01 |
| 1.053 | 51.66 |
| 1.085 | 52 |

In the revised manuscript, the analytical Thevenin-equivalent calculations have been expanded and presented separately for each scenario, allowing the DFCL impedance values to be independently verified. Furthermore, Scenario E has been revised by explicitly modeling the fault-current contribution of the inverter-based PV system. The PV inverter is assumed to operate under the worst-case fault condition with a current-limiting capability of approximately twice its rated current, which is consistent with common inverter protection characteristics. As a result, the calculated short-circuit current, Thevenin equivalent impedance, and DFCL impedance for Scenario E now differ from those of Scenario D. The corresponding discussion has also been expanded to clarify the limited fault-current contribution of inverter-based DGs compared with synchronous and induction generator-based DGs.

**Reviewer’s Comment:**

3. The paper performs only three-phase symmetric fault analysis. Distribution networks that interface with inverter-based DGs commonly experience single-line-to-ground faults as the dominant fault type. The authors should clarify whether the DFCL impedance determined from three-phase analysis is sufficient for unsymmetric faults, or present supplementary analysis for at least single-phase faults, given that the PV inverter's sequence-network behavior during asymmetric faults differs from synchronous machines.

**Author’s Response:**

We sincerely thank the reviewer for this valuable comment. The three-phase symmetrical fault was intentionally selected because it represents the most severe fault condition for evaluating the proposed DFCL and determining its required impedance. Under three-phase faults, the DFCL provides the minimum current-limiting impedance required to maintain proper relay coordination, making it the worst-case design condition.

The proposed DFCL is based on a magnetic-core structure with differentiated phase windings (N, 2N, and 3N), which enables effective operation under both symmetrical and unsymmetrical fault conditions. According to the operating principle of the proposed DFCL and the cited reference, the current-limiting performance under unsymmetrical faults, such as single-line-to-ground faults, is expected to be maintained or further improved because these faults generally produce lower fault-current levels than three-phase faults. In addition, inverter-based PV systems inherently limit their fault current through the converter control strategy, resulting in lower fault-current contribution during asymmetric faults compared with synchronous machines.

Therefore, the DFCL impedance determined from the three-phase fault analysis represents the minimum required limiting impedance. If satisfactory protection coordination is achieved under this worst-case condition, the proposed DFCL is expected to provide equal or better performance under unsymmetrical faults. To clarify this point, an explanatory discussion has been added to the revised manuscript together with the corresponding reference describing the DFCL operating principle.

**Reviewer’s Comment:**

4. The directional detection mechanism of the DFCL is a critical functional requirement of the entire proposal, yet the paper provides no circuit-level description, no detection logic, no response time, and no reference to the specific hardware implementation. Reference [26] (Ghanbari and Farjah, 2013) is cited as the experimental basis, but that paper concerns a unidirectional FCL, not a directional one. The authors must describe how fault current direction is measured (e.g., voltage-polarity comparison, phase angle detection), what the detection latency is, and how false triggers during load switching or motor starting are avoided.

**Author’s Response:**

We sincerely thank the reviewer for this valuable comment. To clarify the operation of the proposed SICFCL, a new section entitled "SICFCL Configuration and Principle of Operation" has been added to Section III. This subsection explains the operating principle of the proposed magnetic-core SICFCL and its directional detection mechanism. The detailed circuit-level implementation, detection logic, and response time incorporated in Section III (lines 185), where the complete hardware implementation was discussed.

**Reviewer’s Comment:**

5. The following references are recommended to consider to strengthen the manuscript's discussion.

[1] E. Dehghanpour, H. K. Karegar, R. Kheirollahi, and T. Soleymani, "Optimal coordination of directional overcurrent relays in microgrids by using cuckoo-linear optimization algorithm and fault current limiter," IEEE Trans. Smart Grid, vol. 9, no. 2, pp. 1365-1375, Mar. 2018, doi: 10.1109/TSG.2016.2587725.

[2] X. Meng, D. Xie, H. Lin, C. Lin, X. Ge and Z. Liu, "Dissipativity-Based Multiport Stability Root-Cause Identification and Mitigation for Solid-State Transformers," in IEEE Transactions on Industrial Electronics, doi: 10.1109/TIE.2026.3658639.

[3] C. Feng, Z. Huang, J. Lin, L. Wang, Y. Zhang and F. Wen, "Aggregation Model and Market Mechanism for Virtual Power Plant Participation in Inertia and Primary Frequency Response," in IEEE Transactions on Power Systems, vol. 41, no. 3, pp. 2101-2117, May 2026, doi: 10.1109/TPWRS.2025.3637538.

**Author’s Response:**

Thank the reviewer for the valuable recommendation. As mentioned above in review (7) We carefully reviewed all the suggested references and evaluated their relevance to the scope of the present study. Since they provide useful background on recent developments related to microgrid protection, fault current limiters, and emerging power-electronic-based power systems, they have been incorporated into the revised manuscript where appropriate to strengthen the literature review and better position the proposed work within the context of recent advances. Specifically:

[1] Dehghanpour et al. (2018), "Optimal coordination of directional overcurrent relays in microgrids by using cuckoo-linear optimization algorithm and fault current limiter," has been cited in the Introduction (Page 4, Lines 82) to support the discussion on directional overcurrent relay coordination and the application of fault current limiters in microgrids.

[2] Meng et al. (2026), "Dissipativity-Based Multiport Stability Root-Cause Identification and Mitigation for Solid-State Transformers," has been cited in the Importance of the fault current limiter (FCL) section (Page 5, Lines 111) to enrich the discussion on power electronic converter-based systems and stability considerations in modern power networks.

[3] Feng et al. (2026), "Aggregation Model and Market Mechanism for Virtual Power Plant Participation in Inertia and Primary Frequency Response," has been cited Importance of the fault current limiter (FCL) section (Page 5, Lines 111) to provide additional background on emerging grid-support technologies and the evolving role of inverter-based resources in modern power system

**Reviewer 2**

**Reviewer’s Comment:**

1- The authors are encouraged to compare their work with recent literature and clearly highlight the novelty beyond Refs. [19] and [25].

**Author’s Response:**

Thank you for your valuable and constructive comment.

We appreciate the reviewer's suggestion to strengthen the comparison with the recent literature and to better emphasize the novelty of the proposed work. During the revision process, we identified that References [19] and [25] were inadvertently duplicated in the reference list. This error has been corrected in the revised manuscript.

Furthermore, we have expanded the literature review by incorporating additional recent studies on fault current limiter (FCL)-based protection strategies for microgrids (see Refs. [15],[16] and [23]. A comparative discussion has been added to clearly distinguish the proposed approach from the existing methods and to better highlight its technical contributions as shown in Table 11.

among the published studies, Refs. [15] and [20] are the closest to the present work since both employ directional fault current limiters to restore overcurrent relay coordination in grid-connected microgrids. Ref. [15] demonstrates the effectiveness of UFCL for maintaining relay coordination; however, it is limited to a specific implementation and does not provide a generalized analytical sizing methodology. Ref. [20] extends this concept by introducing an optimization-based framework that simultaneously optimizes DFCL parameters and system reliability using MCMC and MECPSO/GA algorithms, at the expense of increased computational complexity and reliance on optimization techniques. In contrast, the proposed work eliminates the need for optimization algorithms entirely. Instead, the required DFCL impedance is obtained directly from the network short-circuit level using the MVAsc and Thevenin equivalent impedance, enabling rapid restoration of the original protection coordination without relay resetting or iterative optimization. Moreover, the proposed methodology is applicable to different distributed generation technologies, including synchronous generators, induction generators, and inverter-based DGs, making it more practical for engineering applications.

Table 11. Comparison of the proposed work with Refs. [15],[20]

| Feature | Ref. [15] | Ref. [20] | **Proposed study** |
| --- | --- | --- | --- |
| FCL type | Unidirectional FCL (UFCL) | Directional FCL (DFCL) | Three-phase Saturated Iron-Core DFCL |
| Main objective | Restore OCR coordination | Restore coordination while minimizing reliability and DFCL costs | Restore original protection coordination analytically |
| Methodology | UFCL-based protection scheme | **Optimization-based methodology** (Objective Function + MCMC + MECPSO/GA) | **Analytical methodology (MVAsc + Thevenin)** |
| Optimization algorithm | Not required | **Required** (MECPSO/GA + MCMC reliability evaluation) | **Not required** |
| Relay setting restoration | By UFCL installation | By optimizing DFCL parameters while preserving relay settings | By analytical calculation of the required DFCL impedance without relay resetting |
| Required relay resetting | No | No | No |
| DG integration | General distributed generation | Synchronous generator, PV-based DG, and microgrid operation | Synchronous generator, induction generator, and inverter-based DG |
| Applicability | Specific microgrid configuration | Studied microgrid with predefined scenarios | Applicable to different DG types and ratings |
| Computational complexity | Low | High | **Low** |

**Reviewer’s Comment:**

2- The authors should provide the full mathematical formulation, including decision variables, objective function, constraints, and the solution procedure.

**Author’s Response:**

We sincerely thank the reviewer for this valuable comment. To improve the clarity of the proposed methodology, the mathematical formulation has been expanded in the revised manuscript. The analytical derivation is now presented step by step, starting from the short-circuit capacity (MVAsc) calculation, followed by the determination of the Thevenin equivalent impedance, and finally the analytical calculation of the required DFCL impedance. In addition, the complete calculation sequence has been summarized in a flowchart in comment 4 to facilitate the reproducibility of the proposed approach. Furthermore, as detailed in our response to Reviewer 1 (Comment 2), complete per-scenario calculation steps have been added to the revised manuscript to further improve the transparency and reproducibility of the proposed analytical methodology.

**Reviewer’s Comment:**

3- In Scenario A, the DFCL impedance is obtained from the difference between equivalent Thevenin impedances before and after DG integration. However, the theoretical derivation is insufficient. A detailed mathematical explanation is needed, together with a discussion of its applicability to larger systems with multiple DGs.

We sincerely thank the reviewer for this valuable comment. This observation is closely related to Comment 2, which requested a more comprehensive presentation of the mathematical formulation. Accordingly, the analytical derivation has been expanded in the revised manuscript and is now presented step by step, starting from the short-circuit capacity (MVAsc) calculation, followed by the determination of the Thevenin equivalent impedance, and finally the analytical calculation of the required DFCL impedance. The complete calculation sequence has also been summarized in a flowchart to improve the clarity and reproducibility of the proposed methodology.

In addition, the applicability of the proposed analytical procedure has been further clarified. Since the methodology is based on the equivalent Thevenin impedance seen from the point of common coupling (PCC), it is not restricted to the studied system and can be directly applied to larger power systems with multiple distributed generation units by recalculating the equivalent network impedance for the corresponding operating configuration.

**Reviewer’s Comment:**

4- The proposed approach is referred to as an optimization methodology, yet the paper does not clearly specify whether it relies on analytical calculations, iterative procedures, or optimization algorithms. A flowchart or pseudocode should be included to improve reproducibility.

We sincerely thank the reviewer for this valuable comment. As also addressed in #Reviewer (1), Comment (1), the proposed approach is based on analytical calculations rather than iterative procedures or optimization algorithms. Accordingly, the revised manuscript explicitly clarifies the nature of the proposed methodology and removes any ambiguity regarding the use of optimization techniques.

In addition, following the reviewer's recommendation, a detailed flowchart (Fig. 13) has been incorporated into the manuscript to illustrate in section (4) the complete analytical procedure. The flowchart presents the sequential implementation steps, including input network data, MVAsc calculation, Thevenin equivalent impedance determination, DFCL impedance calculation, ETAP validation, and protection coordination assessment. This addition significantly improves the clarity and reproducibility of the proposed methodology.

Figure 13. Flowchart of the Proposed DFCL Impedance Determination Methodology

**Reviewer’s Comment:**

5.A deeper comparison of fault-current contributions from synchronous generators, induction generators, and inverter-based PV systems would significantly improve the technical insights.

**Author’s Response:**

We sincerely thank the reviewer for this valuable suggestion. To address this comment, the discussion in the Results and Discussion section has been expanded to provide a clearer comparison As shown in Table (12) **:**

The base-case configuration represents the reference operating condition in which the overcurrent relay settings are already properly coordinated; therefore, no DFCL is required. Under this condition, the total fault current at the PCC is **2.076 kA**, while the existing synchronous generator (DG1) contributes **0.198 kA**, corresponding to **9.54%** of the total fault current.

In **Scenario A**, integrating an additional **1.5 MVA synchronous generator (DG2)** increases the DG fault-current contribution to **0.375 kA**, while the total fault current rises to **2.249 kA**. Consequently, a DFCL impedance of **30.01 Ω** is required to restore the original protection coordination. The newly integrated DG contributes **7.87%** of the total fault current.

In **Scenario B**, adding a larger **5 MVA synchronous generator (DG3)** further increases the DG contribution to **0.784 kA**, resulting in a total fault current of **2.657 kA** and a required DFCL impedance of **47.47 Ω**. The new DG contribution reaches **15.39%**, which is the highest among all investigated DG units. This is attributed to the larger generator rating and the inherently high fault-current capability of synchronous generators.

In **Scenario C**, integrating the **1.765 MVA induction generator (WT1)** increases the DG contribution to **0.929 kA**, producing a total fault current of **2.805 kA** with a required DFCL impedance of **50.01 Ω**. However, the newly added induction generator contributes only **5.17%** of the total fault current, which is considerably lower than the contribution of the synchronous generator in Scenario B. This indicates that the induction generator has a lower influence on the fault level than the synchronous generator.

In **Scenario D**, a second induction generator (**WT2**) with a **higher internal impedance** is integrated. Although the overall DG contribution increases to **1.053 kA** and the total fault current reaches **2.932 kA**, the contribution of the newly added DG decreases to **4.23%**. This reduction is mainly attributed to the higher internal impedance of WT2, which limits its fault-current contribution compared with WT1.

Finally, in **Scenario E**, a **1 MVA inverter-based PV system** is added. The total fault current increases slightly to **2.965 kA**, while the overall DG contribution reaches **1.085 kA**, requiring a DFCL impedance of **52 Ω**. Nevertheless, the contribution of the PV unit itself is only **1.08%**, confirming that inverter-based DGs contribute very limited fault current because of their converter current-limiting control.

Overall, the results demonstrate that the total fault current increases progressively with each additional DG integration, leading to a corresponding increase in the required DFCL impedance as shown in Table 13 and Fig 14 from 30.01 Ω in Scenario A to 52 Ω in Scenario E. Furthermore, the fault-current contribution strongly depends on the DG technology and its electrical characteristics. Synchronous generators exhibit the highest contribution, induction generators provide lower contributions, while inverter-based PV systems have the smallest impact on the network fault level.

Table 12. Comparison of Fault-Current Contributions of Different Distributed Generation Technologies

| Scenario | Integrated New DG Type | Integrated DG Rating (MVA) | Fault current contribution from added DGs (KA) | Total Fault current at PCC (KA) | Percentage of fault current contribution from new DG added | DFCL Impedance (Ohm) |
| --- | --- | --- | --- | --- | --- | --- |
| Original Network (DG1) | Synchronous (DG1) | 1.5 | 0.198 | 2.076 | 9.54 % | No need (relays are coordinated) |
| A  (DG1& DG 2) | Synchronous (DG2) | 1.5 | 0.375 | 2.249 | 7.87 % | 30.01 |
| B (DG1& DG 2&DG3) | Synchronous (DG3) | 5 | 0.784 | 2.657 | 15.39 % | 47.47 |
| C  (DG1& DG 2&DG3&WT1) | Induction (WT1) | 1.765 | 0.929 | 2.805 | 5.17 % | 50.01 |
| D  (DG1& DG 2&DG3&WT1&WT2) | Induction (WT2) (different internal impedance) | 1.765 | 1.053 | 2.932 | 4.23 % | 51.66 |
| E  (DG1& DG 2&DG3&WT1&WT2&PV) | PV (with Inverter) | 1 | 1.085 | 2.965 | 1.08 % | 52 |

Table 13. FCL Scenarios impedance vs Short circuit current

| **Scenario** | **A** | **B** | **C** | **D** | **E** |
| --- | --- | --- | --- | --- | --- |
| $\boldsymbol{I}_{\boldsymbol{SC}}$ | 0.375 | 0.784 | 0.929 | 1.053 | 1.085 |
| $\boldsymbol{Z}_{\boldsymbol{FCL}}$ | 30.01 | 47.47 | 50.01 | 51.66 | 52 |

Fig 14. FCL Scenarios impedance vs Short circuit current

| I S.c (KA) | Z fcl (Ohm) |
| --- | --- |
| 0.375 | 30.01 |
| 0.784 | 47.47 |
| 0.929 | 50.01 |
| 1.053 | 51.66 |
| 1.085 | 52 |
