# Supplementary material for: Adaptive and resilient protection coordination in smart microgrids enabled by a directional fault current limiter
Source: PLoS One. 2026 Aug 19;21(8):e0356375. doi: 10.1371/journal.pone.0356375 (PMC13489461; doi:10.1371/journal.pone.0356375)
Supplement: Peer review history

# Peer review: Decision Letter 0

**DOI:** 10.1371/journal.pone.0356375.r001

**Authors:** Zhengmao Li

11 Jun 2026

Dear Dr. M. Hamada,

Thank you for submitting your manuscript to PLOS ONE. After careful consideration, we feel that it has merit but does not fully meet PLOS ONE’s publication criteria as it currently stands. Therefore, we invite you to submit a revised version of the manuscript that addresses the points raised during the review process.

Please submit your revised manuscript by Aug 10 2026 11:59PM. If you will need more time than this to complete your revisions, please reply to this message or contact the journal office at plosone@plos.org. When you're ready to submit your revision, log on to https://www.editorialmanager.com/pone/ and select the 'Submissions Needing Revision' folder to locate your manuscript file.

- A letter that responds to each point raised by the academic editor and reviewer(s). You should upload this letter as a separate file labeled 'Response to Reviewers'.
- A marked-up copy of your manuscript that highlights changes made to the original version. You should upload this as a separate file labeled 'Revised Manuscript with Track Changes'.
- An unmarked version of your revised paper without tracked changes. You should upload this as a separate file labeled 'Manuscript'.

If you would like to make changes to your financial disclosure, please include your updated statement in your cover letter. Guidelines for resubmitting your figure files are available below the reviewer comments at the end of this letter.

If applicable, we recommend that you deposit your laboratory protocols in protocols.io to enhance the reproducibility of your results. Protocols.io assigns your protocol its own identifier (DOI) so that it can be cited independently in the future. For instructions see: https://journals.plos.org/plosone/s/submission-guidelines#loc-laboratory-protocols. Additionally, PLOS ONE offers an option for publishing peer-reviewed Lab Protocol articles, which describe protocols hosted on protocols.io. Read more information on sharing protocols at https://plos.org/protocols?utm_medium=editorial-email&utm_source=authorletters&utm_campaign=protocols.

As the corresponding author, your ORCID iD is verified in the submission system and will appear in the published article. PLOS supports the use of ORCID, and we encourage all coauthors to register for an ORCID iD and use it as well. Please encourage your coauthors to verify their ORCID iD within the submission system before final acceptance, as unverified ORCID iDs will not appear in the published article. *Only*  the individual author can complete the verification step; PLOS staff *cannot*  verify ORCID iDs on behalf of authors.

We look forward to receiving your revised manuscript.

Kind regards,

Zhengmao Li

Academic Editor

PLOS One

Journal Requirements:

When submitting your revision, we need you to address these additional requirements.

1. Please ensure that your manuscript meets PLOS ONE's style requirements, including those for file naming. The PLOS ONE style templates can be found at

https://journals.plos.org/plosone/s/file?id=wjVg/PLOSOne_formatting_sample_main_body.pdf and

https://journals.plos.org/plosone/s/file?id=ba62/PLOSOne_formatting_sample_title_authors_affiliations.pdf

2. Please note that PLOS One has specific guidelines on code sharing for submissions in which author-generated code underpins the findings in the manuscript. In these cases, we expect all author-generated code to be made available without restrictions upon publication of the work. Please review our guidelines at https://journals.plos.org/plosone/s/materials-and-software-sharing#loc-sharing-code and ensure that your code is shared in a way that follows best practice and facilitates reproducibility and reuse.

3. Thank you for stating the following in the Acknowledgments Section of your manuscript:

“This article has been produced with the financial support of the European Union under the REFRESH – Research Excellence For Region Sustainability and High-tech Industries project number CZ.10.03.01/00/22_003/0000048 via the Operational Programme Just Transition.”

We note that you have provided funding information that is not currently declared in your Funding Statement. However, funding information should not appear in the Acknowledgments section or other areas of your manuscript. We will only publish funding information present in the Funding Statement section of the online submission form.

Please remove any funding-related text from the manuscript and let us know how you would like to update your Funding Statement. Currently, your Funding Statement reads as follows:

“The author(s) received no specific funding for this work.”

Please include your amended statements within your cover letter; we will change the online submission form on your behalf.

4. We note that your Data Availability Statement is currently as follows:

“All relevant data supporting the findings of this study are included within the manuscript and its supporting information files. Additional simulation data and analysis results are available from the corresponding author upon reasonable request.”

Please confirm at this time whether or not your submission contains all raw data required to replicate the results of your study. Authors must share the “minimal data set” for their submission. PLOS defines the minimal data set to consist of the data required to replicate all study findings reported in the article, as well as related metadata and methods (https://journals.plos.org/plosone/s/data-availability#loc-minimal-data-set-definition).

For example, authors should submit the following data:

- The values behind the means, standard deviations and other measures reported;

- The values used to build graphs;

- The points extracted from images for analysis.

Authors do not need to submit their entire data set if only a portion of the data was used in the reported study.

If your submission does not contain these data, please either upload them as Supporting Information files or deposit them to a stable, public repository and provide us with the relevant URLs, DOIs, or accession numbers. For a list of recommended repositories, please see https://journals.plos.org/plosone/s/recommended-repositories.

If there are ethical or legal restrictions on sharing a de-identified data set, please explain them in detail (e.g., data contain potentially sensitive information, data are owned by a third-party organization, etc.) and who has imposed them (e.g., an ethics committee). Please also provide contact information for a data access committee, ethics committee, or other institutional body to which data requests may be sent. If data are owned by a third party, please indicate how others may request data access.

5. Please upload a new copy of Figures 2, 4, 5, 6, 8, 9, 10, and 12 as the detail is not clear. Please follow the link for more information:  https://journals.plos.org/plosone/s/figures

6. Please ensure that you refer to Figure 3 in your text as, if accepted, production will need this reference to link the reader to the figure.

7. If the reviewer comments include a recommendation to cite specific previously published works, please review and evaluate these publications to determine whether they are relevant and should be cited. There is no requirement to cite these works unless the editor has indicated otherwise.

**Additional Editor Comments:**

**Please revise**

[Note: HTML markup is below. Please do not edit.]

Reviewers' comments:

Reviewer's Responses to Questions

**Comments to the Author**

1. Is the manuscript technically sound, and do the data support the conclusions?

Reviewer #1: Partly

Reviewer #2: Yes

**********

2. Has the statistical analysis been performed appropriately and rigorously?

Reviewer #1: N/A

Reviewer #2: Yes

**********

3. Have the authors made all data underlying the findings in their manuscript fully available?

The PLOS Data policy

Reviewer #1: Yes

Reviewer #2: Yes

**********

4. Is the manuscript presented in an intelligible fashion and written in standard English?

Reviewer #1: Yes

Reviewer #2: Yes

**********

Reviewer #1: This paper proposes a directional fault current limiter (DFCL) installed at the PCC of a microgrid to restore overcurrent relay coordination after DG integration.

1. The title and abstract claim a "novel optimization methodology" that simultaneously minimizes DFCL installation cost and reliability-related cost. However, no optimization algorithm, no cost model, and no objective function appear anywhere in the main text or in any table or figure. The paper presents a Thevenin-based analytical method for impedance sizing, which is straightforward engineering calculation rather than optimization. The authors should either (a) remove the optimization language and retitle the contribution accurately, or (b) add the full cost model, objective function, and solver details that are referenced in the abstract.

2. The DFCL impedance sizing formula is derived as Z_DFCL = Z_th(base) - Z_th(after DG). For Scenario A the numbers given are 64.51 ohm (base) - 34.22 ohm (after DG2) = 30.29 ohm, which matches Table 11. However, for Scenario B the Thevenin impedance after adding DG3 is listed as 16.57 ohm, yielding Z_DFCL = 47.94 ohm (Table 11), yet Table 12 reports I_SC = 0.784 kA for Scenario B, while Scenario C reports I_SC = 0.929 kA with Z_DFCL = 53.47 ohm, and Scenario D reports I_SC = 1.053 kA with Z_DFCL = 54.65 ohm -- identical to Scenario E. The identical I_SC and Z_DFCL values for Scenarios D and E (Table 12), despite D using an induction WTG and E using a PV inverter whose fault contribution the authors themselves describe as negligible, needs explicit explanation. The paper must show the per-scenario Thevenin calculation steps so readers can verify the numbers independently.

3. The paper performs only three-phase symmetric fault analysis. Distribution networks that interface with inverter-based DGs commonly experience single-line-to-ground faults as the dominant fault type. The authors should clarify whether the DFCL impedance determined from three-phase analysis is sufficient for unsymmetric faults, or present supplementary analysis for at least single-phase faults, given that the PV inverter's sequence-network behavior during asymmetric faults differs from synchronous machines.

4. The directional detection mechanism of the DFCL is a critical functional requirement of the entire proposal, yet the paper provides no circuit-level description, no detection logic, no response time, and no reference to the specific hardware implementation. Reference [26] (Ghanbari and Farjah, 2013) is cited as the experimental basis, but that paper concerns a unidirectional FCL, not a directional one. The authors must describe how fault current direction is measured (e.g., voltage-polarity comparison, phase angle detection), what the detection latency is, and how false triggers during load switching or motor starting are avoided.

5. The following references are recommended to consider to strengthen the manuscript's discussion.

[1] E. Dehghanpour, H. K. Karegar, R. Kheirollahi, and T. Soleymani, "Optimal coordination of directional overcurrent relays in microgrids by using cuckoo-linear optimization algorithm and fault current limiter," IEEE Trans. Smart Grid, vol. 9, no. 2, pp. 1365-1375, Mar. 2018, doi: 10.1109/TSG.2016.2587725.

[2] X. Meng, D. Xie, H. Lin, C. Lin, X. Ge and Z. Liu, "Dissipativity-Based Multiport Stability Root-Cause Identification and Mitigation for Solid-State Transformers," in IEEE Transactions on Industrial Electronics, doi: 10.1109/TIE.2026.3658639.

[3] C. Feng, Z. Huang, J. Lin, L. Wang, Y. Zhang and F. Wen, "Aggregation Model and Market Mechanism for Virtual Power Plant Participation in Inertia and Primary Frequency Response," in IEEE Transactions on Power Systems, vol. 41, no. 3, pp. 2101-2117, May 2026, doi: 10.1109/TPWRS.2025.3637538.

Reviewer #2: The manuscript addresses the application of a directional fault current limiter (DFCL) to restore protection coordination in microgrids with distributed generation. The topic is relevant to modern distribution systems; however, several technical and presentation issues should be addressed。

1.The authors are encouraged to compare their work with recent literature and clearly highlight the novelty beyond Refs. [19] and [25].

2.The authors should provide the full mathematical formulation, including decision variables, objective function, constraints, and the solution procedure.

3.In Scenario A, the DFCL impedance is obtained from the difference between equivalent Thevenin impedances before and after DG integration. However, the theoretical derivation is insufficient. A detailed mathematical explanation is needed, together with a discussion of its applicability to larger systems with multiple DGs.

4.The proposed approach is referred to as an optimization methodology, yet the paper does not clearly specify whether it relies on analytical calculations, iterative procedures, or optimization algorithms. A flowchart or pseudocode should be included to improve reproducibility.

5.A deeper comparison of fault-current contributions from synchronous generators, induction generators, and inverter-based PV systems would significantly improve the technical insights.

**********

what does this mean?). If published, this will include your full peer review and any attached files.

If you choose “no”, your identity will remain anonymous but your review may still be made public.

**Do you want your identity to be public for this peer review?**  For information about this choice, including consent withdrawal, please see our Privacy Policy

Reviewer #1: No

Reviewer #2: No

**********

[NOTE: If reviewer comments were submitted as an attachment file, they will be attached to this email and accessible via the submission site. Please log into your account, locate the manuscript record, and check for the action link "View Attachments". If this link does not appear, there are no attachment files.]

To ensure your figures meet our technical requirements, please review our figure guidelines: https://journals.plos.org/plosone/s/figures

You may also use PLOS’s free figure tool, NAAS, to help you prepare publication quality figures: https://journals.plos.org/plosone/s/figures#loc-tools-for-figure-preparation.

NAAS will assess whether your figures meet our technical requirements by comparing each figure against our figure specifications.

# Author comment: Author response to Decision Letter 1

**DOI:** 10.1371/journal.pone.0356375.r002

17 Jul 2026

Thank you for the valuable comments provided by the editor and reviewers. We have carefully addressed all comments and revised the manuscript accordingly. A detailed point-by-point response, including the corresponding revisions, figures, and tables, is provided in the attached Response to Reviewers document. All changes made in the revised manuscript have been highlighted for clarity.

Attachment Submitted filename: Response to Reviewers.docx

# Peer review: Decision Letter 1

**DOI:** 10.1371/journal.pone.0356375.r003

**Authors:** Zhengmao Li

3 Aug 2026

Adaptive and Resilient Protection Coordination in Smart Microgrids Enabled by a Directional Fault Current Limiter

PONE-D-26-25751R1

Dear Dr. M. Hamada,

We’re pleased to inform you that your manuscript has been judged scientifically suitable for publication and will be formally accepted for publication once it meets all outstanding technical requirements.

Within one week, you’ll receive an e-mail detailing the required amendments. When these have been addressed, you’ll receive a formal acceptance letter and your manuscript will be scheduled for publication.

An invoice will be generated when your article is formally accepted. Please note, if your institution has a publishing partnership with PLOS and your article meets the relevant criteria, all or part of your publication costs will be covered. Please make sure your user information is up-to-date by logging into Editorial Manager at Editorial Manager® and clicking the ‘Update My Information' link at the top of the page. For questions related to billing, please contact billing support.

If your institution or institutions have a press office, please notify them about your upcoming paper to help maximize its impact. If they’ll be preparing press materials, please inform our press team as soon as possible -- no later than 48 hours after receiving the formal acceptance. Your manuscript will remain under strict press embargo until 2 pm Eastern Time on the date of publication. For more information, please contact onepress@plos.org.

Kind regards,

Zhengmao Li

Academic Editor

PLOS One

Additional Editor Comments (optional):

Reviewers' comments:

Reviewer's Responses to Questions

**Comments to the Author**

Reviewer #1: (No Response)

Reviewer #2: All comments have been addressed

**********

2. Is the manuscript technically sound, and do the data support the conclusions?

Reviewer #1: (No Response)

Reviewer #2: Yes

**********

3. Has the statistical analysis been performed appropriately and rigorously?

Reviewer #1: (No Response)

Reviewer #2: Yes

**********

4. Have the authors made all data underlying the findings in their manuscript fully available?

The PLOS Data policy

Reviewer #1: (No Response)

Reviewer #2: Yes

**********

5. Is the manuscript presented in an intelligible fashion and written in standard English?

Reviewer #1: (No Response)

Reviewer #2: Yes

**********

Reviewer #1: (No Response)

Reviewer #2: (No Response)

**********

what does this mean?). If published, this will include your full peer review and any attached files.

If you choose “no”, your identity will remain anonymous but your review may still be made public.

**Do you want your identity to be public for this peer review?**  For information about this choice, including consent withdrawal, please see our Privacy Policy

Reviewer #1: No

Reviewer #2: No

**********

# Editor report: Acceptance letter

**DOI:** 10.1371/journal.pone.0356375.r004

**Authors:** Zhengmao Li

PONE-D-26-25751R1

PLOS One

Dear Dr. M. Hamada,

I'm pleased to inform you that your manuscript has been deemed suitable for publication in PLOS One. Congratulations! Your manuscript is now being handed over to our production team.

At this stage, our production department will prepare your paper for publication. This includes ensuring the following:

* All references, tables, and figures are properly cited

* All relevant supporting information is included in the manuscript submission,

* There are no issues that prevent the paper from being properly typeset

You will receive further instructions from the production team, including instructions on how to review your proof when it is ready. Please keep in mind that we are working through a large volume of accepted articles, so please give us a few days to review your paper and let you know the next and final steps.

Lastly, if your institution or institutions have a press office, please let them know about your upcoming paper now to help maximize its impact. If they'll be preparing press materials, please inform our press team within the next 48 hours. Your manuscript will remain under strict press embargo until 2 pm Eastern Time on the date of publication. For more information, please contact onepress@plos.org.

You will receive an invoice from PLOS for your publication fee after your manuscript has reached the completed accept phase. If you receive an email requesting payment before acceptance or for any other service, this may be a phishing scheme. Learn how to identify phishing emails and protect your accounts at https://explore.plos.org/phishing.

If we can help with anything else, please email us at customercare@plos.org.

Thank you for submitting your work to PLOS ONE and supporting open access.

Kind regards,

PLOS ONE Editorial Office Staff

on behalf of

Dr Zhengmao Li

Academic Editor

PLOS One
